# Altered trafficking of Kv1-Kvβ2 leads to neuronal hyperexcitability and memory deficits in amyloid-β pathology

**DOI:** 10.1186/s13024-026-00936-2

**Published:** 2026-03-10

**Authors:** Young-Sin Cho, Seo-Hyun Kim, Shin-Hyeon Ryu, Chaelin Chung, Nahyun Lee, Namhoon Kim, Muhah Jeong, Youngwon Kim, Jimin Gwak, Se-Young Choi, Takaomi Saido, Yong-Keun Jung

**Affiliations:** 1 School of Biological Sciences, Seoul National University, 1 Gwanak-ro, Gwanak-gu, Seoul, 08826 Republic of Korea; 2 Department of Neurology, Institute for Cell Engineering, Johns Hopkins University School of Medicine, Baltimore, MD 21205 USA; 3 Department of Physiology, Dental Research Institute, Seoul National University School of Dentistry, Seoul, 03080 Korea; 4 Interdisciplinary Program in Neuroscience, Seoul National University, Seoul, 08826 Korea; 5 Laboratory for Proteolytic Neuroscience, RIKEN Center for Brain Science, Saitama, 3510198 Japan

**Keywords:** Amyloid-β pathology, Alzheimer’s disease, Intracellular amyloid-β, Voltage-gated potassium channel β2 (Kvβ2), APP^NL−G−F^ knock-in mouse, Neuronal excitability, Cognitive function

## Abstract

**Background:**

Potassium channel dysfunction and altered neuronal excitability potentially contribute to cognitive decline in amyloid-β (Aβ) pathology. In particular, the accumulation of intracellular Aβ, preceding extracellular plaque deposition, has been implicated in dysregulated excitability and early-stage neuronal stress. However, the molecular mechanisms linking intracellular Aβ to altered neuronal excitability remain incompletely understood. This study aimed to identify a mediator of intracellular Aβ toxicity contributing to neuronal dysfunction in Aβ pathology.

**Methods:**

We performed a genome-wide human protein chip assay, followed by in vitro binding assays, surface plasmon resonance, and in vivo colocalization analysis using postmortem Alzheimer’s disease (AD) brain tissues in which Aβ pathology is observed. We generated neuronal cell lines stably expressing mRFP-Aβ to model intracellular Aβ accumulation and investigate its pathogenic effects. In addition, we evaluated potassium channel activity and neurotoxicity in Aβ-treated neuronal cells and assessed neuronal excitability and cognitive functions in APP^NL−G−F^ knock-in mice using electrophysiological recordings and behavioral tests.

**Results:**

Voltage-gated potassium channel β2 (Kvβ2) was identified as a novel intracellular Aβ-binding protein. Aβ directly bound to the N-terminus of Kvβ2, disrupting its interaction with EB1 and impairing Kv1-Kvβ2 localization to the axon initial segment. In neuronal cell lines expressing intracellular mRFP-Aβ, Kvβ2 colocalized with Aβ in the cytosol, and Kv1 trafficking to the plasma membrane was markedly reduced. Consistent with this observation, colocalization of Aβ and Kvβ2 was confirmed in the brains of AD patients. In addition, enforced expression of Kvβ2, but not Aβ-binding-defective Kvβ2 ∆(2–18) mutant, rescued potassium channel dysfunction and suppressed neurotoxicity in Aβ-treated neuronal cells. Furthermore, lentiviral delivery of Kvβ2 into APP^NL−G−F^ mice reduced neuronal hyperexcitability, rescued activity-related neuronal markers, and ameliorated memory deficits, whereas the Kvβ2 ∆(2–18) mutant did not.

**Conclusion:**

These findings suggest that the interaction between Kvβ2 and Aβ mediates neuronal hyperexcitability and memory impairment in APP^NL−G−F^ mice, elucidating a potential mechanism underlying Kv1 channel dysfunction in Aβ pathology.

**Supplementary Information:**

The online version contains supplementary material available at 10.1186/s13024-026-00936-2.

## Background

Amyloid-β (Aβ) pathology is recognized as a driver of neuronal dysfunction and represents a core early feature of Alzheimer’s disease (AD), encompassing a broad spectrum of biochemical and cellular abnormalities arising from Aβ misfolding, accumulation, and aggregation [1, 2]. Although the neurotoxic effects of extracellular Aβ through its interactions with various receptors, such as prion protein, NMDAR, FcγRIIb, and RAGE, have been extensively characterized [3–6], emerging evidence suggests that intracellular Aβ also plays a critical role in AD pathogenesis [7, 8]. Intracellular Aβ often originates by the release from endosomal compartments during its production or cellular uptake from extracellular space [9], accumulating within various subcellular compartments, including the trans-Golgi network, endoplasmic reticulum, endosomes, lysosomes, and mitochondria [10, 11]. Its levels strongly correlate with neuronal loss and cognitive decline, particularly in early-stage AD [12]. In addition, accumulating studies suggest that intracellular proteins mediate Aβ neurotoxicity via their malfunctions. Mitochondrial-localized Aβ was shown to bind to alcohol dehydrogenase, triggering organelle dysfunction and contributing to neuronal damage and cognitive deficits [13, 14]. Vacuolar-type H^+^-ATPase (V-ATPase), a key regulator of endolysosomal acidification, binds to Aβ to impair lysosomal activity [8], leading to membrane permeabilization and leakage of Aβ into the cytosol and thereby exacerbating neuronal toxicity [9, 15]. Despite these findings, the full spectrum of Aβ-binding intracellular proteins and their pathological consequences remain poorly understood.

The voltage-gated potassium channels (Kv) play a pivotal role in regulating neuronal excitability by controlling action potential repolarization and firing frequency [16]. Kv-family proteins consist of multiple subfamilies, such as Kv1 to Kv12 [17], each interacting with specific auxiliary β-subunits that critically regulate channel properties and membrane trafficking [18]. Numerous studies have implicated the dysfunction of Kv channels in Aβ pathology. Several subtypes, such as Kv3.1, Kv7.2, and Kv7.3, are downregulated in the AD brain [19, 20]. In addition, Kv3.4 and Kv4.2 have been implicated in Aβ-induced neuronal death and neurotoxicity, respectively, primarily due to their expressional alterations in Aβ pathology [21, 22]. While Kv1.3 is upregulated in microglia and involved in neuroinflammation in AD patients [23], the pathological role and dysfunction of Kv1 in neurons and Aβ pathology have been largely unexplored. Especially, the functional disruption of Kv1 caused directly by Aβ remains poorly understood.

In this study, we screened intracellular target proteins of Aβ using a genome-wide human recombinant protein chip assay and identified Kvβ2, an auxiliary subunit that regulates the trafficking of Kv1 complexes, including Kv1.2, to the axon initial segment (AIS) by interacting with end-binding protein 1 (EB1) [24]. Aβ-binding to Kvβ2 impaired the Kvβ2-EB1 interaction, thereby disrupting Kv1 channel trafficking to cell surface. In APP^NL−G−F^ mice, which exhibit Aβ pathology, ectopic expression of Kvβ2, but not Aβ-binding-defective mutant, ameliorated neuronal hyperexcitability and memory deficits, uncovering a mechanism underlying the Aβ-mediated dysfunction of Kv1 channel in Aβ pathology.

## Methods

### Preparation of synthetic Aβ oligomers

Recombinant Aβ_1−42_ (rPeptide, A-1163) was pretreated with hexafluoroisopropanol (HFIP), dissolved in DMSO (2 mM), and diluted in PBS (pH 7.4) to 125 µM. After incubation at 4 ℃ for 48 h, the solution was centrifuged (13,400 × *g*, 10 min) and the supernatant containing soluble oligomers was collected and stored at -80 ℃ [8, 25].

### Human protein chip assay screening for Aβ-binding proteins

A human proteome chip (HuProt™ v3.1, CDI Labs), printed on 75 mm × 25 mm SuperEpoxy 3 glass slides (ArrayIt Corp.) and containing over 20,000 purified full-length human recombinant proteins, was blocked with 2% BSA in PBST for 2 h at room temperature and incubated with Alexa Fluor 532-labeled Aβ (3 µg) at 4 ℃ for 8 h. After washing and rinsing, the array was dried and scanned using a microarray laser scanner. Following background correction and removal of non-specific signals, the top 1% of proteins based on affinity scores (A-scores) were selected as potential Aβ-binding candidates. Intracellular proteins with A-scores exceeding 10,000 were then selected for data presentation (GENEON BIOTECH, Daejeon, Republic of Korea) [8].

### Antibodies

The following antibodies were used for Western blot, immunoprecipitation (IP), immunocytochemistry and immunohistochemistry. 6E10 (Biolegend, 803003); Aβ (β-Amyloid Rabbit mAb, ABclonal, A26342 for analyzing tissue samples); Kvβ2 (Origene, TA331456 for immunostaining; Neuromab, 75 − 021 for Western blot and co-IP); MAP2 (Biolegend, 822501); Ankyrin G (Santa Cruz Biotechnology, sc-12719); EB1 (Santa Cruz Biotechnology, sc-47704; ABclonal, A2614); Kv1.2 (ABclonal, A6295); c-Fos (Abcam, AB190289); NeuN (Merck, MAB377); VGLUT1 (ABclonal, A17188); PSD95 (Cell Signaling Technology, 3409); HA (Santa Cruz Biotechnology, sc-7392); His (Cell Signaling Technology, 2365); V5 (Abcam, ab27671); FLAG (Sigma, F1804); RFP (MBL, PM005); GFP (Santa Cruz Biotechnology, sc-9996); α-Tubulin (Santa Cruz Biotechnology, sc-23948); HRP-Streptavidin (Sigma, RABHRP3); normal mouse IgG (Santa Cruz Biotechnology, sc-3877) and normal rabbit IgG (Cell Signaling Technology, 2729 S) antibodies were used as controls.

### Western blot

Western blot was performed as previously described [8]. Proteins from cell lysates or mouse hippocampal extracts were separated by SDS-PAGE and transferred to PVDF membranes using a semi-dry transfer system. Membranes were blocked with 5% BSA in TBS-T (10 mM Tris-Cl, pH 8.0, 150 mM NaCl, 0.5% Tween-20) and then incubated with primary and HRP-conjugated secondary antibodies. Protein bands were visualized using enhanced chemiluminescence.

### Immunocytochemistry

Cells were fixed with 4% paraformaldehyde (PFA), washed with PBS. For permeabilized conditions, cells were incubated in blocking buffer containing 1% BSA and 0.05% saponin (Sigma, 47036) for 15 min at room temperature prior to antibody staining. After overnight incubation at 4 ℃ with primary antibodies against Kvβ2 (1:300, Origene) and Ankyrin G (1:50), cells were incubated with appropriate fluorophore-conjugated secondary antibodies. For immunocytochemical analysis using anti-HA antibody (1:200) in the non-permeabilized condition, cells were fixed with PFA without exposure to saponin and surface-localized YFP-Kv1.2-HA was visualized [26]. Images were acquired using a Zeiss LSM700 confocal microscope.

### Immunohistochemistry of AD patient brain

Immunohistochemistry was performed on paraffin-embedded frontal cortex sections (rectus gyrus) isolated from an AD patient as previously described with minor modifications [8]. Antigen retrieval was conducted using 70% formic acid for 30 min at room temperature. Sections were blocked with 5% BSA and incubated overnight at 4 ℃ with primary antibodies against Kvβ2 (1:250, Origene), 6E10 (1:500), and MAP2 (1:1000). Corresponding fluorophore-conjugated secondary antibodies were applied, and images were acquired using a Zeiss LSM700 confocal microscope. Fluorescence intensity histograms were generated using Zen software.

### Comparative analysis of *KCNA2* and *KCNAB2* levels in the brains of AD patients

Publicly available gene expression datasets were used to examine mRNA levels of *KCNA2* and *KCNAB2* in AD and control human brain tissues. Microarray data were obtained from the Gene Expression Omnibus (GEO) under the accession numbers GSE1297 (hippocampal CA1 region; control, *n* = 9; AD, *n* = 22) and GSE4757 (entorhinal cortex; control, *n* = 9; AD, *n* = 10) [27, 28]. Raw expression data were downloaded and normalized expression values were extracted for *KCNA2* and *KCNAB2*.

### DNA constructs

Tag-fused constructs were generated by PCR-based cloning or site-directed mutagenesis. His-tagged Kvβ2 was cloned into pET-28a using *Not*I/*Xho*I sites. A construct lacking the N-terminal His tag was generated from His-Kvβ2-His by site-directed mutagenesis. GST-tagged EB1 was cloned into pGEX-4T2 using *Sma*I/*Not*I, and a monomeric red fluorescent protein (mRFP)-tagged Aβ was inserted into mRFP-C1 using *Xho*I/*Hin*dIII. For C-terminal tagging, V5-TurboID sequence was PCR-amplified and inserted into Kvβ2 using *Xba*I/*Bam*HI. A green fluorescent protein (GFP)- and FLAG-tagged Kvβ2 constructs were cloned into GFP-C1 and p3xFLAG-CMV-10, respectively, using *Eco*RI/*Bam*HI. For lentiviral expression, Kvβ2 wild type (WT) and Δ(2–18) mutant were subcloned into pLVX-puro vector (Clontech, 632164) using *Eco*RI/*Bam*HI. Deletion (Δ) mutants (ΔN, ΔC, Δ(2–18)) were generated by site-directed mutagenesis. YFP-Kv1.2-HA was a kind gift from Dr. C Gu (Ohio State University, USA) [26].

### Purification of bacterially expressed proteins

GST-tagged EB1 and His-tagged Kvβ2 were expressed in *E. coli* BL21 cells cultured in LB medium containing either ampicillin or kanamycin. Protein expression was induced with 0.5 mM IPTG at 16 ℃ for 16 h when cultures reached OD_600_ ≈ 0.6. Cells were lysed by sonication in lysis buffer (20 mM Tris-HCl, 500 mM NaCl, pH 8.0), with 2 mM imidazole for His-tagged proteins. After centrifugation, the supernatant was incubated with Ni-NTA agarose (His-Kvβ2 protein) or glutathione-Sepharose 4B (GST-EB1 protein) overnight at 4 ℃ and bound proteins were eluted using lysis buffer containing either 0.5 M imidazole or 5 mM reduced glutathione. For tag removal, GST-EB1 was digested with thrombin protease (1 unit/µg, Cytiva, 27-0846-01), and residual GST was removed by re-binding to glutathione resin.

### Surface plasmon resonance (SPR)

SPR analysis was performed on a Biacore system (Cytiva, Marlborough, MA, USA) using an NTA sensor chip. The surface was conditioned with 350 mM EDTA and primed with 0.5 mM NiCl_2_ prior to capturing His-Kvβ2 proteins. After equilibration, Aβ was injected into running buffer and binding kinetics were monitored. Regeneration was performed using 350 mM EDTA. Sensorgrams were fitted to a 1:1 Langmuir binding model using Biacore software to determine the dissociation constant (KD).

### Co-immunoprecipitation (Co-IP) assay

Co-IP was performed as previously described with minor modifications [8]. Cells or mouse hippocampal tissues were lysed with IP lysis buffer containing 25 mM Tris-Cl (pH 7.5), 15 mM NaCl, 0.5% Triton X-100, 0.25% sodium deoxycholate and 1:100 protease inhibitor cocktail (Quartett, QTPPI1012). Lysates were centrifuged at 13,400 × *g* for 10 min at 4 ℃ and the resulting supernatants were incubated with appropriate primary antibody (2 µg) overnight at 4 ℃. Antibody-protein complexes were captured using Protein G Sepharose (GE Healthcare, 17-0618-05), washed four times with PBS, and analyzed by Western blot. For in vitro Aβ- binding assays, lysates were incubated with Aβ (1 µM), pre-cleared with Protein G Sepharose, and then incubated with primary antibody (2 µg) overnight at 4 ℃. Subsequent IP steps were performed as described above.

### Generation of mRFP-Aβ-expressing HT22 cells and assessment of Aβ aggregation

Mouse hippocampal HT22 cells were transfected with mRFP-Aβ and selected with puromycin (1 µg/ml). To assess Aβ solubility, cells were lysed in TBS (20 mM Tris-Cl, pH 7.4, 150 mM NaCl) by repeated passage through a 26-gauge syringe and lysates were subjected to ultracentrifugation at 100,000 × *g* for 1 h at 4 ℃. Supernatant was collected as soluble Aβ fraction. Pellet was resuspended in 5 M guanidine hydrochloride and incubated for 12 h at 4 ℃ to extract insoluble Aβ. After a second centrifugation, the guanidine-soluble fraction was collected for Western blot [29].

To evaluate the aggregation of intracellular Aβ, mRFP-Aβ-expressing HT22 cells were treated with methylene blue (1 or 5 µM, Sigma, M4159) for 24 h. Aggregation was assessed by two approaches. First, biochemical fractionation was performed and guanidine-soluble Aβ was analyzed by Western blot. Second, cells were fixed with 4% PFA, counterstained with Hoechst 33342 and imaged using a Zeiss LSM700 confocal microscope to visualize the distribution and aggregation of mRFP-Aβ. To quantify puncta size and number, images were analyzed using ImageJ [30]. An identical intensity threshold was applied across all groups to segment puncta from background, followed by binary conversion and quantification using ‘Analyze Particles’ function. Size and circularity filters were used to exclude background artifacts, and the same parameters were applied uniformly to all images.

### Cell surface biotinylation assay

Cell surface biotinylation was performed as previously described with minor modifications [31]. The mRFP-Aβ-expressing and control HT22 cells were incubated with sulfo-NHS-SS-biotin (1 mg/ml, APExBIO, A8005) in PBS for 30 min at 4 ℃ to label surface proteins. After washing with ice-cold PBS, cells were lysed in IP lysis buffer and clarified by centrifugation at 12,000 × *g* for 10 min at 4 ℃. Supernatants were incubated with streptavidin agarose resin for 4 h at 4 ℃. Beads were washed with PBS and analyzed by Western blot.

### Flux OR potassium ion channel assay

Flux OR assay was performed as previously described [32]. HT22 cells stably expressing Kvβ2 WT or Δ(2–18) mutants were pretreated with Aβ (5 µM) for 30 h prior to dye loading. Cells were incubated with Flux OR dye in loading buffer for 60 min at room temperature in the dark. Thallium influx was induced by adding stimulus buffer containing 2.4 mM thallium sulfate and real-time fluorescence (Ex/Em = 490/525 nm) was recorded every 4 s for 5 min using a fluorescence plate reader.

### Assessment of cell viability

HT22 cells stably expressing Kvβ2 WT or Δ(2–18) mutant were treated with Aβ (5 µM) for 48 h to assess cell viability. Live and dead cells were visualized by calcein-AM and propidium iodide (PI) staining, respectively, and imaged using a fluorescence microscope. The percentage of dead cells was calculated based on the number of PI-positive versus total (calcein-AM-positive) cells. In parallel, cell viability was quantified using MTT assay. After Aβ treatment, cells were incubated with MTT solution (0.5 mg/ml, Sigma, M-2003) for 3 h at 37 ℃ and formazan was solubilized in DMSO. Absorbance was measured at 570 nm using a microplate reader.

### APP^NL−G−F^ knock-in mice

APP^NL−G−F^ knock-in mice harbor humanized Aβ sequence with three familial Alzheimer’s disease (FAD) mutations: the Swedish (KM670/671NL), Beyreuther/Iberian (I716F), and Arctic (E693G) mutations. APP^NL−G−F^ mice were obtained from the RIKEN Center for Brain Science, Japan [33] and Age-matched C57BL/6J WT mice were used as controls.

### Intracerebroventricular injection of lentivirus

Intracerebroventricular (ICV) injection was performed as described previously [8, 34]. Lentiviruses (titer: 1 × 10^8^ TU/ml) carrying either Kvβ2 WT or the Δ(2–18) mutant, as well as control empty lentiviruses prepared in the same manner, were delivered using a Hamilton microliter 702 syringe (Hamilton Company, 80400) equipped with a 26-gauge needle.

### Electrophysiological recordings

Mice were anesthetized with isoflurane and decapitated. Brains were rapidly extracted and sectioned into 300 μm thick slices in ice-cold, oxygenated dissection buffer (bubbled with 95% O_2_ / 5% CO_2_) containing (in mM): 5 KCl, 1.23 NaHPO_4_, 26 NaHCO_3_, 212.7 sucrose, 10 MgCl_2_, and 0.5 CaCl_2_. Slices containing hippocampus were prepared and recovered for 40 min in oxygenated artificial cerebrospinal fluid (ACSF) composed of (in mM): 124 NaCl, 5 KCl, 1.23 NaHPO_4_, 26 NaHCO_3_, 1 MgCl_2_, and 2 CaCl_2_. For recording, the perfusion solution consisted of ACSF supplemented with picrotoxin (100 µM). Recording pipettes (4–8 MΩ resistance) were filled with the following internal solutions: for spontaneous excitatory postsynaptic current (sEPSC), excitability, and single action potential recordings, evoked EPSC- (in mM) 135 K-gluconate, 8 NaCl, 10 HEPES, 2 Na_2_ATP, and 0.2 Na_3_GTP. For evoked EPSC recordings, a stimulus electrode was placed in the Schaffer’s collateral pathway, and the stimulus was delivered by isolated current source (SIU9A). Neurons were voltage-clamped at -60 mV, and the stimulus intensity was adjusted to evoke maximal EPSC responses. Input resistance, access resistance, and resting membrane potential were continuously monitored. Recordings were performed using a Multiclamp 700 A amplifier (Molecular Devices). Cells were excluded from analysis if input or access resistance changed by more than 20% during the recording. Signals were low-pass filtered at 3 kHz and digitized at 10 kHz using a Digidata 1440 A digitizer (Molecular Devices).

### Behavior tests

Behavior tests were performed as described previously [8, 25].

#### Y-maze

Mice were placed at the end of one arm and allowed to explore the maze (32.5 cm × 15 cm) freely for 7 min. An arm entry was counted when the entire body entered an arm and spontaneous alternation behavior was calculated as the percentage of triads in which the mouse entered three different arms consecutively, using the formula; spontaneous alteration (%) = [number of alternations / (total arm entries − 2)] × 100.

#### Passive avoidance

This test consisted of habituation (Day 1), training (Day 2), and testing (Day 3). Mice were placed in a two-compartment apparatus (40 × 20 × 20 cm) consisting of a lit and a dark chamber. During training, a mild foot shock (0.25 mA, 2 s) was delivered upon full entry into the dark compartment. Latency to enter the dark compartment was recorded on the test day (cutoff: 7 min).

#### Novel object recognition

The novel object recognition (NOR) test included two days of habituation, followed by training and two testing days. Mice explored the open-field arena (22 × 27 × 30 cm) for 7 min per session. During testing, one of the familiar objects was replaced with a novel object. The discrimination ratio was calculated as the time spent exploring the novel object divided by the total time exploring both objects.

### Statistical analysis

All statistical analyses were performed using GraphPad Prism 10 software (GraphPad Software, CA, USA) and figures were assembled using Adobe Photoshop 2025 (Adobe, CA, USA). Sample sizes and a few replicates are indicated in the corresponding figure legends. Statistical significance was assessed using an unpaired two-tailed student’s t-test for comparisons between two groups, one-way ANOVA for comparisons among three or more groups with a single independent variable or two-way ANOVA for experiments involving two independent variables with Bonferroni post-hoc analysis. *P* values were obtained from two-tailed statistical tests.

## Results

### Genome-wide identification of Kvβ2 as an oligomeric Aβ-binder

**Fig. 1:**
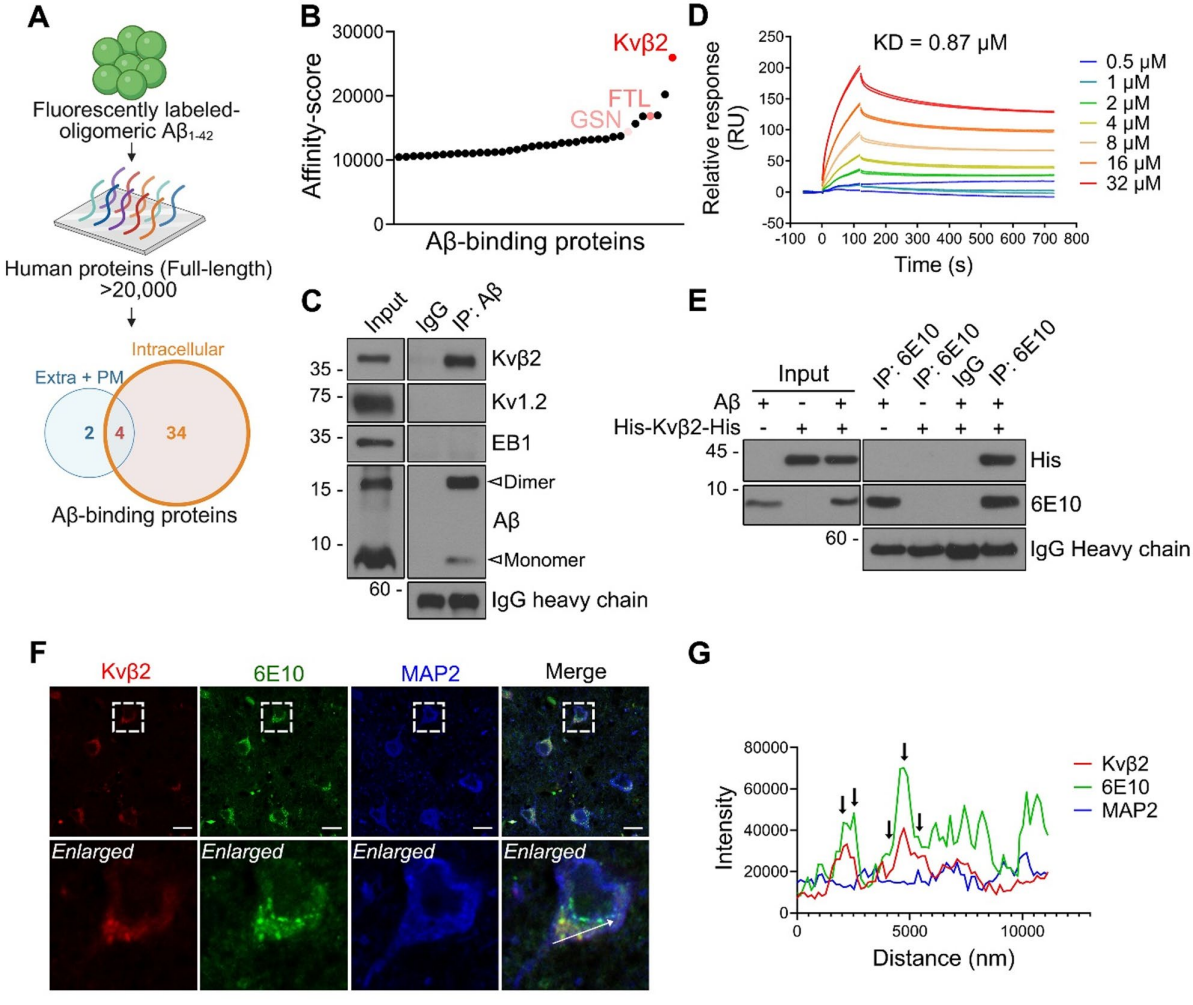
Kvβ2 binds to oligomeric Aβ in vitro and in AD samples. **A**,** B** A protein chip binding assay was performed by incubating fluorescence-labeled oligomeric Aβ with a library of 20,000 human recombinant proteins. Among the Aβ-binding proteins, intracellular proteins were identified **(A)**. PM, Plasma membrane. A graph represents the putative intracellular Aβ-interactors sorted by affinity-score **(B)**. FTL (Ferritin light chain) and GSN (gelsolin) are shown as known Aβ-binding proteins. **C** The hippocampal lysates of 9-month-old APP^NL−G−F^ mice were subjected to IP assay using anti-Aβ (ABclonal) antibody, followed by Western blot analysis. **D** Purified Kvβ2-His proteins immobilized on a sensor chip NTA were incubated with the indicated concentrations of Aβ and analyzed for the binding with SPR. **E** Purified His-Kvβ2-His protein (1 µM) was incubated with Aβ (1 µM) at 4 ℃ for 6 h and subjected to co-IP assay using 6E10 antibody. **F**,** G** Paraffin sections of the frontal lobe (rectus gyrus) from patients with AD were immunostained with anti-Kvβ2 (Origene), 6E10, and anti-MAP2 antibodies, and MAP2-positive cells were examined by fluorescence microscopy. The white line indicates the region of interest used for line-scan fluorescence intensity analysis to assess colocalization **(F)**. The fluorescent intensities of Kvβ2, 6E10, and MAP2 on the line in the enlarged images were measured using Zen software and positions showing coincident peaks in the intensity profiles were marked by arrows **(G)**. Scale bars, 10 μm **(F)**. 10% of the total cell lysate was loaded as input **(C**,** E)**

To identify novel proteins that bind to oligomeric Aβ, we performed a protein chip assay employing more than 20,000 purified human recombinant proteins. From this screening, we identified proteins that bound to the fluorescently labeled oligomeric Aβ on the protein chip. We defined the proteins within the top 1% of A-scores (163 proteins, Supplementary Material 2) as potential Aβ-binding candidates and further analyzed the subset of intracellular proteins with A-scores above 10,000 (38 proteins). Among the putative high-affinity interactors, most were intracellular proteins rather than extracellular proteins. (Fig. 1A). Especially, Kvβ2 showed the highest affinity score among the 38 intracellular Aβ-binding proteins, including ferritin light chain [35] and gelsolin [36] (Fig. 1B), and was thus characterized for the cellular binding. We first examined the interaction between Kvβ2 and Aβ in the brain tissues of APP^NL−G−F^ mice, which exhibit physiologically relevant Aβ pathology without overexpression artifacts and show a progressive increase in the hippocampal oligomeric Aβ deposition from 2.5 to 12 months of age, accompanied by synaptic impairment observed at 6–8 months [33, 37, 38]. IP assays using Aβ antibody of the hippocampal lysates prepared from 9-month-old APP^NL−G−F^ mice showed that Aβ bound to Kvβ2, but not to Kv1.2 or EB1 (Fig. 1C). Interestingly, Kvβ2 exhibited binding to both Aβ monomers and dimers (Supplementary Fig. 1A).

Using SPR analysis, we further characterized the interaction between purified recombinant Kvβ2 protein and oligomeric Aβ. Results revealed that Aβ bound directly to His-Kvβ2 protein immobilized on an NTA chip in a dose-dependent manner, showing a binding affinity with a dissociation constant (KD) of 0.87 µM (Fig. 1D). In addition, we confirmed this interaction between purified His-Kvβ2 protein and Aβ with an in vitro binding assay (Fig. 1E). With the immunohistochemical assay of AD patients’ tissues, we further assessed the in vivo interaction between Kvβ2 and intracellular Aβ. Kvβ2 colocalized with the 6E10-positive Aβ within neurons in the postmortem frontal lobe and hippocampus of AD patients (Fig. 1F, G and Supplementary Fig. 1B-D). These results indicate that Kvβ2 interacts and colocalizes with Aβ in the brain tissues under pathological conditions.

### Aβ impairs neuronal trafficking of Kv1 via disrupting the Kvβ2-EB1 interaction

**Fig. 2:**
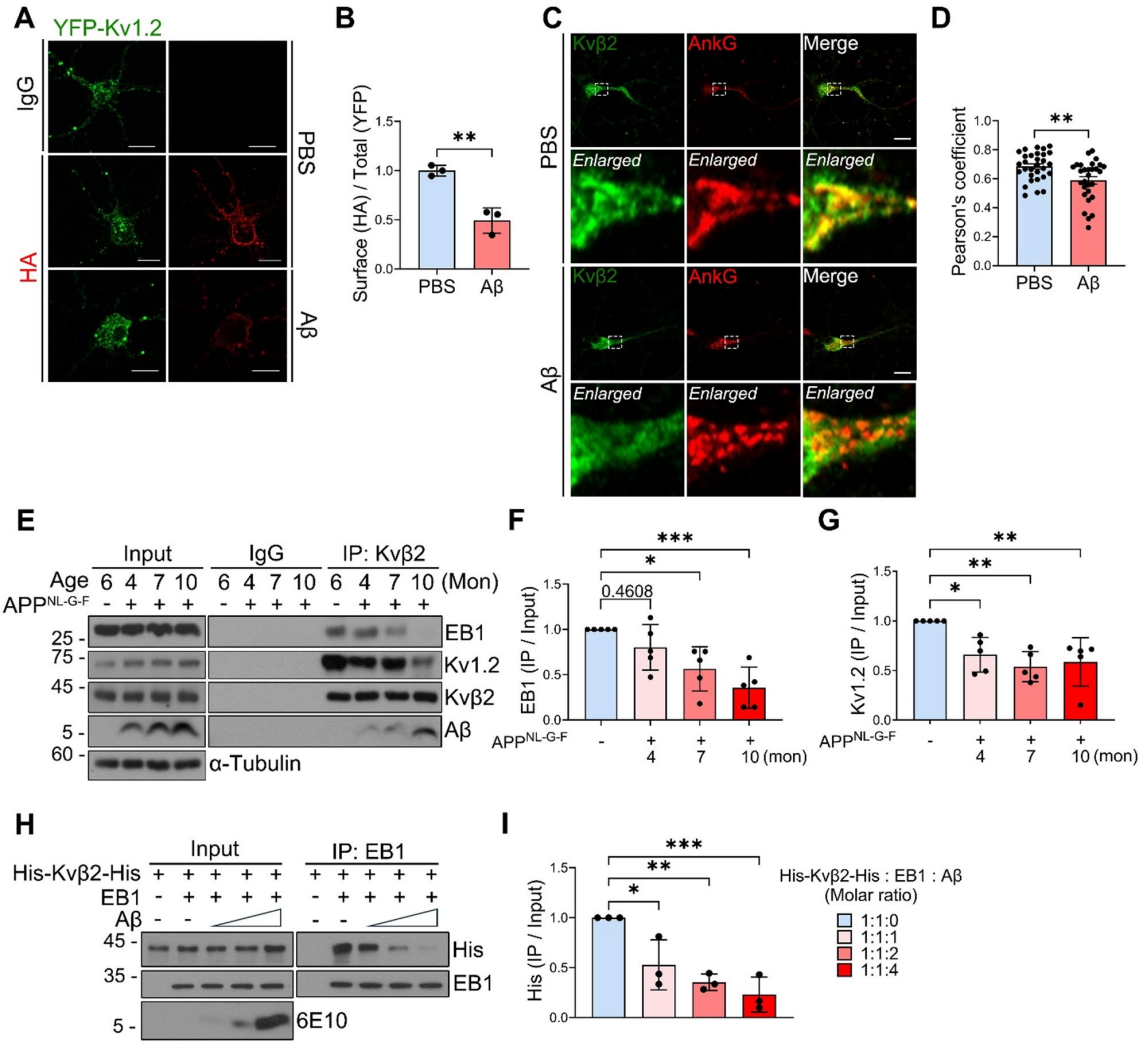
Aβ interferes with the Kvβ2-EB1 interaction and reduces surface localization of Kv1 channels. **A**,** B** Primary cortical neurons (Days in vitro [DIV] 8–10) were transduced with lentiviruses carrying YFP-Kv1.2-HA for 72 h and treated with Aβ (5 µM) for additional 30 h. After immunostaining using anti-HA antibody without permeabilization, cells were observed under fluorescence microscope **(A)**. The relative ratios of Kv1.2-HA signal on cell surface to total level (YFP-Kv1.2) **(B)** were quantified using ImageJ (*n* = 3). **C**,** D** Primary hippocampal neurons (DIV 12–15) were treated with Aβ (5 µM) for 36 h and immunostained with anti-Kvβ2 (Origene) and anti-AnkG antibodies and analyzed by confocal microscope **(C)**. Pearson’s coefficients were calculated to assess Kvβ2 and Ank-G colocalization (*n* = 30 cells for PBS-treated and *n* = 29 for Aβ-treated) **(D)**. **E-G** Hippocampal lysates of 4-, 7-, and 10-month-old APP^NL−G−F^ mice and 6-month-old WT mice were subjected to IP assay using anti-Kvβ2 (Neuromab) antibody **(E)**. The relative ratios of EB1 **(F)** and Kv1.2 **(G)** to their input on the blots were quantified using ImageJ (*n* = 5). **H**,** I** Purified His-Kvβ2-His proteins, EB1 proteins, and Aβ were incubated in the following molar ratios (His-Kvβ2-His: EB1: Aβ = 1:1:0, 1:1:1, 1:1:2 and 1:1:4) and subjected to an in vitro binding and IP assays using anti-EB1 antibody **(H)**. The signals of His-Kvβ2-His on the blots were quantified and normalized by input (*n* = 3) **(I)**. Bars represent mean ± S.D. Unpaired *t*-test, two-tailed **(B**,** D)** and One-Way ANOVA with Bonferroni post-hoc analysis **(F**,** G**,** I)**. *P < 0.05, **P < 0.01, ***P < 0.001. Scale bars, 10 μm (**A**,** C**). 10% of the total cell lysate was loaded as input (**E**,** H**)

When we analyzed the expression levels of *KCNAB2* and *KCNA2*, which encode Kvβ2 and Kv1.2, respectively, in the hippocampus and entorhinal cortex using publicly available transcriptomic datasets from AD patients, we found no significant differences of them between AD and control samples (Supplementary Fig. 2A). Given that Kvβ2 has been shown to regulate the trafficking of Kv1 channels to the AIS in neurons [24] and binds to Aβ, we investigated whether Aβ affects the localization of Kv1 channels at the neuronal membrane. To address this, we used YFP-fused Kv1.2-HA construct which contains both YFP at the N-terminus and HA tag in the first extracellular domain of Kv1.2 (Supplementary Fig. 2B). With YFP fluorescence imaging and immunostaining assays using HA antibody, we could differentiate cell surface-localized YFP-Kv1.2-HA from total cellular YFP-Kv1.2-HA (Fig. 2A). Intriguingly, compared to control cells, Aβ treatment markedly reduced levels of cell surface-localized YFP-Kv1.2-HA (relative ratios of HA immunoreactivity to total fluorescence) and increased YFP-Kv1.2 signals inside neuronal cell body in primary cortical neurons (Fig. 2A, B and Supplementary Fig. 2C). Immunocytochemical analysis using anti-ankyrin G (Ank G) antibody, a marker of the AIS, revealed that Aβ treatment significantly impaired the AIS-targeting of Kvβ2 in primary hippocampal neurons (Fig. 2C, D).

As Kvβ2 mediates axonal targeting of Kv1 channels by interacting with the microtubule plus-end tracking protein EB1 [24, 26], we next investigated whether the Kvβ2-Aβ interaction interferes with the formation of Kvβ2-EB1 complex. Structural prediction using AlphaFold suggests that Aβ-binding to Kvβ2 alters the predicted interaction mode between Kvβ2 and EB1, prompting us to verify this possibility by IP analysis (Supplementary Fig. 2D, E). Therefore, we assessed the interaction between Kvβ2 and EB1 in the hippocampus of APP^NL−G−F^ mice. We first examined intraneuronal Aβ distribution in the hippocampus by MAP2 staining in 4- and 7-month-old APP^NL−G−F^ mice. We observed 6E10 signals in the MAP2-positive neurons at 4 months and these signals increased further with aging, accompanied by prominent extracellular plaques (Supplementary Fig. 3A-C). Consistently, the Kvβ2-Aβ interaction was detectable in 4- and 7-month-old APP^NL−G−F^ mice, whereas the extent of this interaction markedly increased at 10 months (Supplementary Fig. 3D). In 4-month-old APP^NL−G−F^ mice, which do not show severe Aβ pathology yet, levels of the Kvβ2-EB1 complex showed a tendency to be lower than those in 6-month-old control mice (Fig. 2E, F). However, levels of the Kvβ2-EB1 complex were reduced in the hippocampus of 7-month-old APP^NL−G−F^ mice and were largely reduced in 10-month-old APP^NL−G−F^ mice (Fig. 2E, F). In addition, the age-dependent decrease in the Kv1.2-Kvβ2 complex in APP^NL−G−F^ inversely correlated with the increase of Aβ levels (Fig. 2E, G and Supplementary Fig. 3E). In these mice, levels of Kv1.2, EB1, and Kvβ2 were not changed during aging (Supplementary Fig. 3F-H).

Furthermore, we performed an in vitro competition binding assay using purified proteins Kvβ2, EB1, and Aβ. Intriguingly, when we added the Aβ into the reactions containing Kvβ2 and EB1 proteins, the interaction between Kvβ2 and EB1 decreased in an Aβ concentration-dependent manner (Fig. 2H, I). Together, these findings suggest that Aβ disrupts the formation of the Kv1-Kvβ2-EB1 complex.

### Aβ-binding-defective Kvβ2 mutant fails to protect against Aβ neurotoxicity

**Fig. 3:**
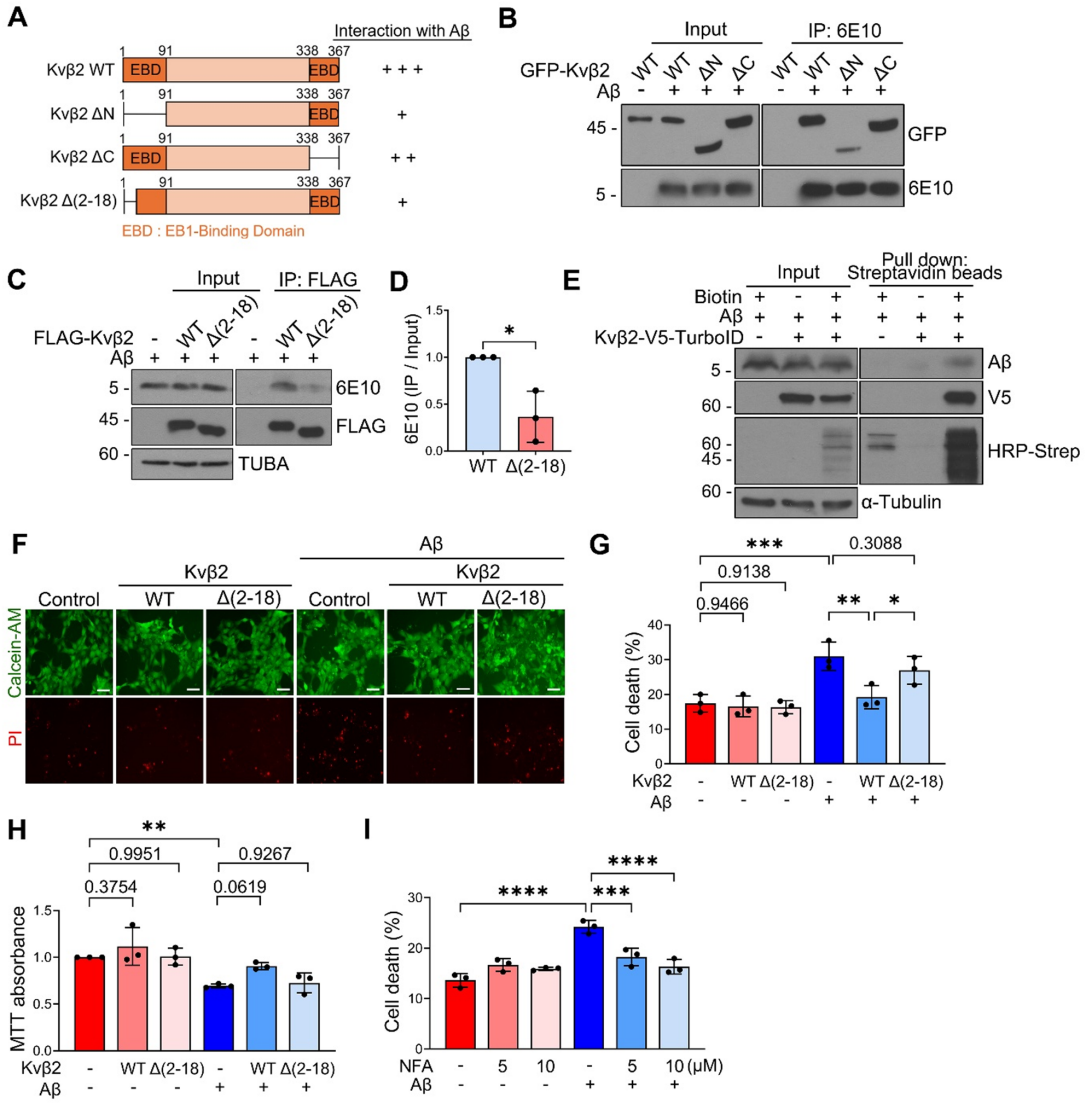
Deletion of Aβ-binding region in Kvβ2 abolishes its protection against Aβ neurotoxicity. **A** Schematic representation of Kvβ2 deletion mutants and their interaction with Aβ. A greater number of ‘+’ symbols reflect relatively stronger interaction. **B** HEK293T cells were transfected with GFP-Kvβ2 WT or deletion mutants for 24 h and cell lysates were incubated with Aβ (1 µM) for 6 h, followed by IP assay using 6E10 antibody. **C**,** D** Lysates of HEK293T cells transfected with FLAG-Kvβ2 WT or Δ(2–18) mutant were incubated with Aβ (1 µM) and then subjected to IP assay using anti-FLAG antibody **(C)**. Signals on the blots were quantified using ImageJ and normalized by input **(D)**. **E** HT22 cells were transfected with Kvβ2-V5-TurboID for 24 h, followed by Aβ (5 µM) treatment for 30 h. After incubation with 100 µM biotin for 4 h, biotinylated proteins were pulled down using streptavidin beads and subjected to Western blot. **F-H** HT22 cells stably expressing Kvβ2 WT or Δ(2–18) mutant were treated with Aβ (5 µM) for 48 h, followed by staining with Calcein-AM (1 µM) and PI (1 µg/ml) **(F**,** G)** or by an MTT assay **(H)**. Scale bars, 10 μm. **I** HT22 cells were treated with the indicated concentrations of NFA alone or together with Aβ (5 µM) for 42 h and stained with Calcein-AM (1 µM) and PI (1 µg/ml). Bars represent mean ± S.D. Unpaired *t*-test, two-tailed **(D)**, Two-Way ANOVA with Bonferroni post-hoc analysis **(G**,** H**,** I)**. *P < 0.05, **P < 0.01, ***P < 0.001, ****P < 0.0001. **(G)**. *n* = 3. 10% of the total cell lysate was loaded as input **(B**,** C**,** E)**

Since the Aβ-Kvβ2 binding disrupts the interaction between Kvβ2 and EB1, we decided to determine the specific region within Kvβ2 responsible for binding to Aβ. Given that the EB1-binding regions of Kvβ2 reside within amino acids 1–90 and 339–367 [26], we generated Kvβ2 mutants lacking these regions (ΔN and ΔC) and examined their binding ability to Aβ (Fig. 3A). IP analysis in HEK293T cells revealed that the GFP-Kvβ2 ΔN mutant exhibited markedly reduced binding to Aβ compared to the GFP-Kvβ2 WT and ΔC mutant (Fig. 3B), indicating that the N-terminal region of Kvβ2 largely contributes to its binding to Aβ. We then generated a smaller N-terminal deletion mutant, lacking amino acids 2–18, and performed IP analysis (Fig. 3A). We found that Kvβ2 Δ(2–18) mutant showed weaker Aβ-binding than Kvβ2 WT did (Fig. 3C, D), identifying that the region spanning amino acids 2–18 in Kvβ2 is required for Aβ binding and suggesting potential overlap with the EB1-binding region.

To investigate the functional significance of the Kvβ2-Aβ interaction, we generated HT22 cells stably expressing either Kvβ2 WT or Δ(2–18) mutant at comparable levels (Supplementary Fig. 4A). Using the TurboID assay, which allows the detection of a temporal binding state of proximal proteins through the biotinylation [39], we found that exogenously applied Aβ was labeled with biotin by Kvβ2-TurboID in HT22 cells (Fig. 3E). This result indicates that Aβ is internalized into cells and binds to intracellular Kvβ2. We then performed a Flux OR reporter assay to measure potassium channel activity in these stable cells following Aβ treatment [32]. Intriguingly, Aβ treatment impaired Flux OR reporter activity (Supplementary Fig. 4B). However, this impairment was significantly mitigated by the ectopic expression of Kvβ2 WT, but not by Kvβ2 Δ(2–18) mutant (Supplementary Fig. 4B). Since the impairment of potassium channels contributes to neuronal death of hippocampal neurons [40, 41], we addressed whether the Kvβ2-Aβ interaction affected Aβ neurotoxicity in HT22 cells. We found that Aβ-induced neurotoxicity was suppressed in HT22 cells expressing Kvβ2 WT, but not in cells expressing the Kvβ2 Δ(2–18) mutant, as evidenced by PI staining (Fig. 3F, G) and MTT assays (Fig. 3H). Further, treatment with niflumic acid (NFA), a Kv1 channel activator [42], significantly suppressed Aβ neurotoxicity in HT22 cells (Fig. 3I), supporting the role of Kv1 channel activity in Aβ neurotoxicity. Collectively, these findings suggest that the Kvβ2-Aβ interaction contributes to Kv1 channel activity and Aβ neurotoxicity.

### Kvβ2 co-localizes with intracellular Aβ and regulates surface localization of Kv1 channel

**Fig. 4:**
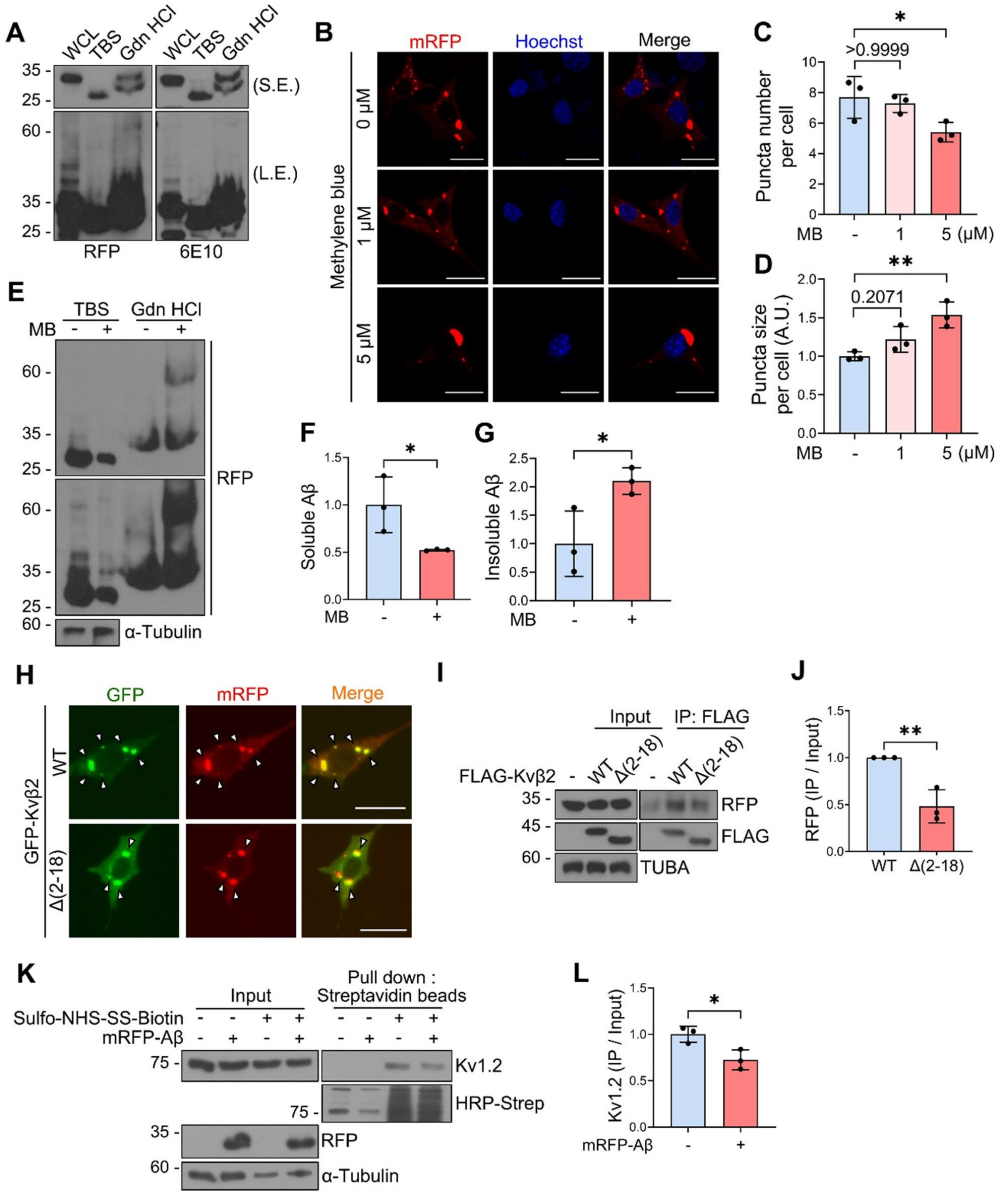
Kvβ2 colocalizes with mRFP-Aβ aggregates, interfering with cell surface localization of Kv1.2. **A** HT22 cell lysates expressing mRFP-Aβ were fractionated into TBS- and 5 M guanidine-HCl-soluble (Gdn HCl) fractions through ultracentrifugation, followed by Western blot using anti-RFP and 6E10 antibodies. S.E., short exposure, L.E., long exposure. **B-D** HT22/mRFP-Aβ cells were treated with methylene blue (MB, 1 or 5 µM) for 24 h and observed under fluorescence microscope **(B)**. The numbers **(C)** and sizes **(D)** of RFP-positive puncta per cell (Total 104–132 cells per group) were quantified using ImageJ. A.U., arbitrary unit. **E-G** After treatment of HT22/mRFP-Aβ cells with MB (5 µM) for 24 h, TBS- and guanidine-HCl-soluble fractions were analyzed by Western blot **(E)**. The signals of soluble **(F)** and insoluble **(G)** mRFP-Aβ on the blots were quantified by ImageJ. **H** HT22/mRFP-Aβ cells were transfected with GFP-Kvβ2 WT or Δ(2–18) mutant for 24 h and observed under fluorescence microscope. Arrowheads indicate colocalization of GFP-Kvβ2 and mRFP-Aβ. **I**,** J** HT22/mRFP-Aβ cells were transfected with FLAG-Kvβ2 WT or Δ(2–18) mutant for 24 h, followed by IP assay using anti-FLAG antibody **(I)**. The relative ratios of the immunoprecipitated mRFP-Aβ to input on the blots were measured with ImageJ **(J)**. **K**,** L** HT22 control and HT22/mRFP-Aβ cells were treated with sulfo-NHS-SS-biotin (1 mg/ml) and subjected to pull-down assay using streptavidin beads, followed by Western blot **(K)**. The levels of biotinylated Kv1.2 on the blots were quantified using ImageJ and normalized by input **(L)**. Bars represent mean ± S.D. Unpaired *t*-test, two-tailed **(F**,** G**,** J**,** L)**, One-Way ANOVA with Bonferroni post-hoc analysis **(C**,** D).** *P < 0.05, **P < 0.01. *n* = 3. Scale bars, 10 μm (**B**,** H**). 10% of the total cell lysate was loaded as input **(I**,** K)**

Previous studies on intracellular oligomeric Aβ have predominantly employed APP mutant overexpression or knock-in models [43, 44]. We thus decided to visualize and confirm whether Kvβ2 interacts with cellular and aggregated forms of Aβ and have generated HT22 cells stably expressing mRFP-tagged Aβ_1−42_ (HT22/mRFP-Aβ). With a guanidine HCl-fractionation assay, we characterized the biochemical properties of cellular mRFP-Aβ and observed both guanidine HCl-soluble and insoluble Aβ species within cells, indicative of intracellular mRFP-Aβ aggregate formation (Fig. 4A). Consistent with this, we also detected puncta forms of cytosolic mRFP-Aβ of heterogeneous sizes (Fig. 4B). In addition, we characterized cellular mRFP-Aβ puncta using methylene blue (MB), a compound previously reported to reduce oligomeric Aβ levels while promoting fibrillar Aβ species formation [45]. MB treatment led to a significant reduction in the number of mRFP-Aβ puncta, accompanied by an increase in their size (Fig. 4B-D). Furthermore, guanidine HCl fractionation analysis revealed a decrease of soluble Aβ species and an increase of insoluble species (Fig. 4E-G), showing an inverse correlation between puncta size and guanidine HCl solubility.

Using these cells, we investigated whether Kvβ2 associates with intracellular aggregates of Aβ. Examination of subcellular distribution showed extensive colocalization of GFP-Kvβ2 with mRFP-Aβ puncta (Fig. 4H) and a similar pattern was observed in primary hippocampal neurons (Supplementary Fig. 4C), while GFP-Kvβ2 Δ(2–18) mutant predominantly colocalized with larger mRFP-Aβ puncta but not with small mRFP-Aβ puncta (Supplementary Fig. 4D). To quantitatively assess the interaction between Kvβ2 and mRFP-Aβ, we performed IP analysis and found that mRFP-Aβ bound to FLAG-Kvβ2 (Fig. 4I, J). On the other hand, the interaction between mRFP-Aβ and FLAG-Kvβ2 Δ(2–18) mutant was significantly reduced. With a cell surface biotinylation assay [31], we assessed whether the interaction between Kvβ2 and mRFP-Aβ alters the membrane trafficking of Kv1 channels. Using membrane-impermeable Sulfo-NHS-SS-biotin, we found that cell surface localization of Kv1 channel was reduced in the mRFP-Aβ-expressing HT22 cells compared to control cells (Fig. 4K, L). These findings indicate that the intracellular Aβ aggregates bind to Kvβ2 and interfere with cell membrane trafficking of Kv1 channels, leading to reduced surface localization.

### Kvβ2 expression ameliorates neuronal hyperexcitability and cognitive impairment in APP^NL−G−F^ mice

**Fig. 5:**
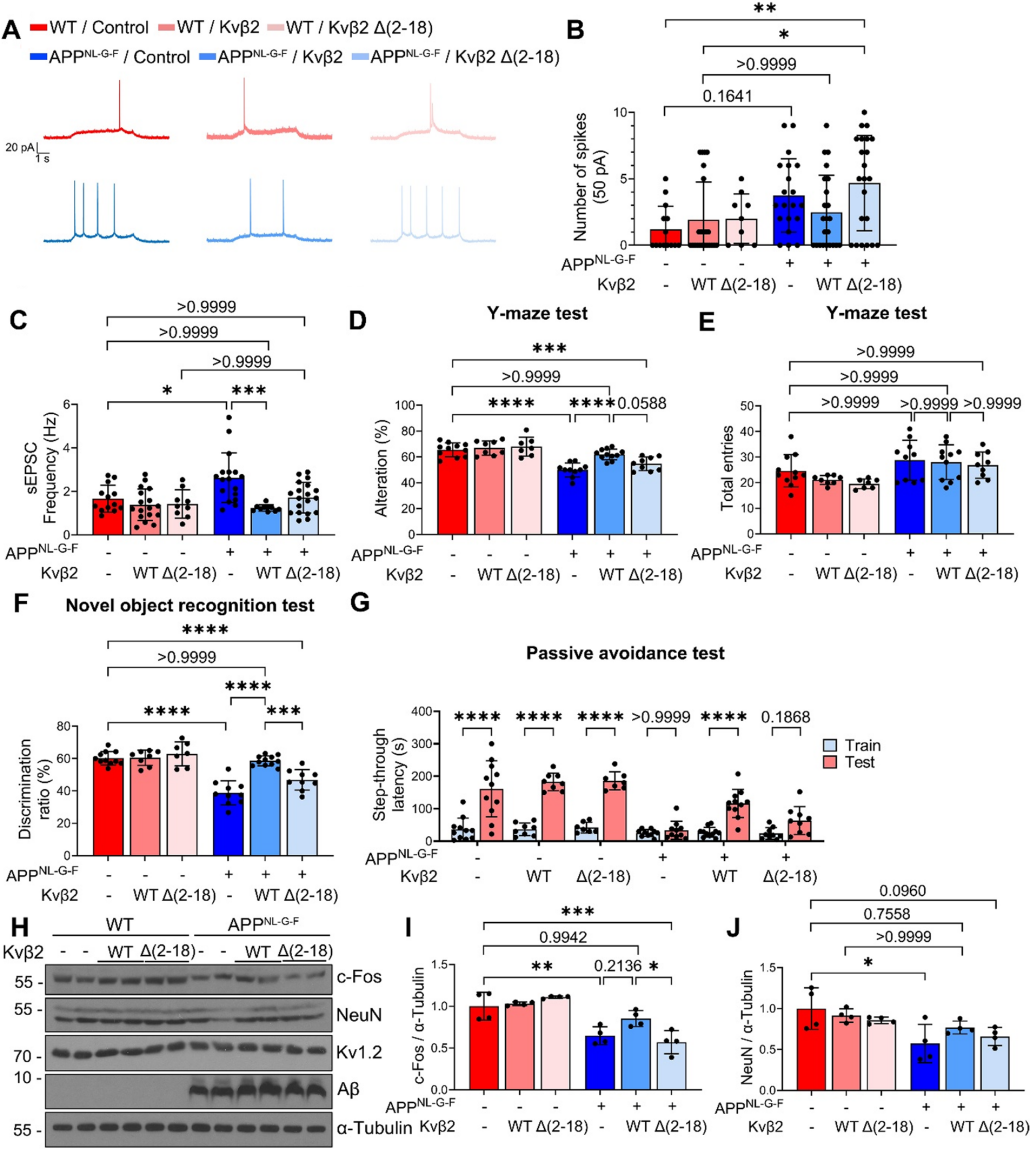
Lentiviral expression of Kvβ2 attenuates neuronal hyperexcitability and memory deficits in APP^NL−G−F^ mice. **A** Representative trace of neuronal excitability in the CA1 hippocampal pyramidal neurons at 50 pA. Scale bars: 1 s, 20 pA. **B** Spike counts were measured in response to 50 pA current injection. WT/Control: *n* = 14 cells, WT/Kvβ2 WT: *n* = 23 cells, WT/Kvβ2 Δ(2–18): *n* = 9 cells, APP^NL−G−F^/Control: *n* = 20 cells, APP^NL−G−F^/Kvβ2 WT: *n* = 23 cells and APP^NL−G−F^/Kvβ2 Δ(2–18) mutant: *n* = 22 cells. **C** The hippocampal tissues slices were assessed for electrophysiological recordings to measure sEPSC frequency. WT/Control: *n* = 13 cells, WT/Kvβ2 WT: *n* = 17 cells, WT/Kvβ2 Δ(2–18): *n* = 9 cells, APP^NL−G−F^/Control: *n* = 17 cells, APP^NL−G−F^/Kvβ2 WT: *n* = 10 cells, APP^NL−G−F^/Kvβ2 Δ(2–18): *n* = 19 cells. **D-G** 7-month-old mice were analyzed Y-maze test **(D**,** E)**, novel object recognition test **(F)**, and passive avoidance test **(G)**. WT/Control: *n* = 11, WT/Kvβ2 WT: *n* = 8, WT/Kvβ2 Δ(2–18): *n* = 7, APP^NL−G−F^/Control: *n* = 10, APP^NL−G−F^/Kvβ2 WT: *n* = 11, APP^NL−G−F^/Kvβ2 Δ(2–18): *n* = 9. **H-J** After electrophysiological recordings, hippocampal tissue lysates of 5.5- to 6-month-old mice were subjected to Western blot **(H)**. The levels of c-Fos **(I)** and NeuN **(J)** on the blots were quantified using ImageJ and normalized by α-Tubulin (*n* = 4). Bars represent mean ± S.D. Two-way ANOVA with Bonferroni post-hoc analysis **(B**,** C**,** D**,** E**,** F**,** G**,** I**,** J)**. *P < 0.05, **P < 0.01, ***P < 0.001, ****P < 0.0001

Aberrant neuronal excitability in the hippocampus has been observed in 2- to 4-month-old APP^NL−G−F^ mice with a possible contribution to disease pathophysiology [37, 46, 47]. We thus assessed whether the neuroprotective effects of Kvβ2 against Aβ neurotoxicity might rescue the neuronal hyperexcitability in APP^NL−G−F^ mice. APP^NL−G−F^ mice were ICV injected with lentiviruses carrying either Kvβ2 WT or Δ(2–18) mutant at 3 months of age and analyzed using electrophysiological recordings at 5.5 months (Supplementary Fig. 5A). Electrophysiological recording assays showed neuronal hyperexcitability in the hippocampal CA1 pyramidal neurons of APP^NL−G−F^ mice compared to age-matched control mice (Fig. 5A, B and Supplementary Fig. 5B). Intriguingly, the numbers of spikes in APP^NL−G−F^ mice were reduced nearly to the levels observed in age-matched control mice by Kvβ2 WT expression, while Kvβ2 Δ(2–18) mutant failed to exhibit this reduction (Fig. 5A, B and Supplementary Fig. 5C). In WT mice, expression of either Kvβ2 WT or Δ(2–18) mutant did not affect spike firing, with no differences observed across groups (Fig. 5A, B and Supplementary Fig. 5D).

In addition, we assessed synaptic proteins in WT and APP^NL−G−F^ mice expressing Kvβ2 WT or Kvβ2 Δ(2–18) mutant. Immunohistochemical analysis revealed that VGLUT1 and PSD95 levels in the hippocampal CA1 region were comparable across those groups (Supplementary Fig. 6A-D). Accordingly, we examined synaptic transmission via eEPSC and sEPSC recordings. The eEPSC amplitudes did not differ among the groups, whereas sEPSC analysis revealed preserved synaptic efficacy but an increased frequency of spontaneous events (Supplementary Fig. 6G, H and Fig. 5C). Notably, sEPSC frequency in APP^NL−G−F^ mice was prevented from increasing by Kvβ2 WT expression (Fig. 5C). These results indicate that Kvβ2 overexpression mitigates neuronal hyperexcitability via its N-terminal domain in APP^NL−G−F^ mice.

Given that abnormal neuronal excitability is implicated in contributing to cognitive dysfunction in Aβ pathology model mice [48], we addressed whether the mitigation of hyperexcitability by Kvβ2 could improve cognitive function in APP^NL−G−F^ mice. Since APP^NL−G−F^ mice exhibit memory deficits at 6 to 8 months of age [33, 49], mice were delivered with lentiviruses expressing Kvβ2 WT or Δ(2–18) mutant at 5 months of age and analyzed for their behaviors at 7 months (Supplementary Fig. 7A). Y-maze tests showed spatial memory impairment in APP^NL−G−F^ mice compared to age-matched control mice, which was significantly attenuated by the expression of Kvβ2 WT but not by Kvβ2 Δ(2–18) mutant (Fig. 5D). In the Y-maze test, total entries of the mice did not differ among groups (Fig. 5E). This protective effect of Kvβ2 was also observed in the recognition memory (Fig. 5F) and aversive memory (Fig. 5G), as assessed via novel object recognition and passive avoidance tests, respectively. Again, these memory impairments were not rescued by the expression of Kvβ2 Δ(2–18) (Fig. 5F, G). Thus, the increased expression of Kvβ2 WT alleviates memory impairment in APP^NL−G−F^ mice, probably through suppressing Aβ pathology.

These mice brains were analyzed biochemically for pathologies associated with these behaviors. Western blot analysis showed that the levels of c-Fos, a marker of neuronal activity, and NeuN, a marker of mature neurons, were reduced in the hippocampus of 6-month-old APP^NL−G−F^ mice compared to control mice (Fig. 5H). Interestingly, the reduction of c-Fos and NeuN was prevented by Kvβ2 WT expression but not by Kvβ2 Δ(2–18) mutant in APP^NL−G−F^ mice (Fig. 5I, J). Consistently, NeuN immunostaining in the hippocampal CA1 region revealed no significant differences in the numbers of NeuN-positive neurons across groups. On the other hand, NeuN signal intensity was reduced in APP^NL−G−F^ mice compared with WT mice but not much reduced in APP^NL−G−F^ mice expressing Kvβ2 (Supplementary Fig. 6C, E, F). In contrast, the levels of Kv1.2 and Aβ were comparable across groups (Supplementary Fig. 7B, C). We also confirmed comparable lentivirus-mediated expression of Kvβ2 WT and Δ(2–18) mutant in the brains of each group (Supplementary Fig. 7D, E). These results show a correlation between memory function and activity-related neuronal markers in the brains of experimental groups of APP^NL−G−F^ mice. Taken together, we propose that Aβ binds to Kvβ2 to cause the dysfunction of Kv1 channel, leading to neurotoxicity and eventually contributing to learning and memory impairment in Aβ pathology models (Supplementary Fig. 7F).

## Discussion

In the human protein chip assay, we identified Kvβ2 as along with a number of intracellular Aβ-binding proteins, such as ferritin light chain and gelsolin which were previously shown to bind to Aβ to modulate its aggregation [36] and neurotoxicity [35]. When interpreting these screening results, several technical aspects of the HuProt™ microarray should be considered. Protein immobilization and conformational constraints on the array surface may differentially affect the accessibility of Aβ-interacting epitopes and yeast-expressed membrane proteins may not fully adopt their native structures, which could account for a relative enrichment of intracellular Aβ interactors. While several Aβ-binding proteins have been previously identified, their functions are largely confined to modulating Aβ aggregation, regulating inflammatory responses, or influencing cellular energy metabolism [50, 51]. Especially, few Aβ-interacting proteins have been reported to directly affect neuronal excitability, a hallmark feature of early AD. In contrast, Aβ binding to Kvβ2 impairs the intracellular trafficking of Kv1 channels, providing a mechanistic insight into a disrupted axonal excitability in the affected neurons, which further underlies cognitive decline in Aβ pathology.

Alterations in AIS resulting from cytoskeletal disruption have been observed in several tauopathy model systems [52, 53]. Tau has also been implicated in modulating the distribution of key AIS components, including EB proteins [54]. Notably, It was shown that tau V337M mutant binds to EB3, leading to neuronal dysfunction in frontotemporal dementia [55]. EB3 plays critical roles in maintaining AnkG stability at the AIS and coordinating interactions between actin and microtubule cytoskeletons [56, 57], while EB1 contributes to the axonal transport of Kv1 channels and the regulation of neuronal excitability [26, 58]. Given that tau regulates the localization of EB3 [59, 60] and that EB3 organizes stable microtubule tracks toward AIS [57], it is conceivable that Aβ and tau may synergistically contribute to disruptions in neuronal excitability.

Our findings demonstrate a direct intracellular interaction between Kvβ2 and Aβ. However, it is also reasonable to consider extracellular Aβ signaling through cell-surface receptors, such as prion protein, LRP1, and NMDA, initiating signaling pathways that affect neuronal functions [61–63]. A recent study demonstrated that Aβ inhibits a potassium channel in a prion protein-dependent manner [64]. Although extracellular Aβ was administered in a subset of our experiments, the observed effects are linked to intracellular events, as evidenced by two distinct experimental findings. First, using our TurboID-based proximity labeling system, we detected an interaction between extracellularly treated Aβ and intracellular Kvβ2, indicating that Aβ can access and engage intracellular targets. Extracellular Aβ can enter neurons through multiple routes, including receptor-mediated endocytosis via LRP1, RAGE, PrPC, or α7-nAChR and also micropinocytosis [65]. Once internalized, as we have shown, Aβ primarily traffics through the endo-lysosomal system, where it perturbs vesicular transport and lysosomal acidification [8, 66]. Disruption of the lysosomal integrity may potentially allow Aβ to leak into the cytosol [67]. Consistent with this model, cellular expression of the viral protease Nia, which degrades oligomeric Aβ, reduces mitochondrial Aβ accumulation and neurotoxicity [68], suggesting that a portion of extracellar Aβ can reach the cytosol before engaging intracellular organelles. Second, in a system where mRFP-Aβ was expressed intracellularly, we observed impaired trafficking of Kv1 channels. We have previously reported that the internalized Aβ is crucial for Aβ neurotoxicity [8, 25]. Accordingly, both extracellular and intracellular Aβ may communicate in ways that contribute to neurotoxicity, deserving further important investigation into this potential interplay.

In our study, Aβ interferes with the axonal targeting of Kv1 channels by binding to Kvβ2. In addition to its role in Kv1 channel trafficking, Kvβ2 also associates with Kv4 channels, influencing their expression and functional properties [69, 70]. Kv4 channels are largely expressed in somatodendritic membrane, where they contribute to the attenuation of dendritic excitatory inputs and the regulation of local signal integration [24, 71]. Conversely, Kv1 channels are primarily localized to axonal compartments and involved in setting action potential thresholds and facilitating membrane repolarization [24]. Moreover, Kvβ2 preferentially associates with Kv1 channels in the brain and binds more efficiently to Kv1 than to Kv4 [72, 73], allowing a more prominent role of Kvβ2 in regulating Kv1 channel localization and function.

Kvβ2 functions as a modulatory subunit of Kv1 channels, which are key regulators of neuronal excitability [74]. Interestingly, the expression of Kvβ2 enhanced electrophysiological properties and improved cognitive function and mitigated the reduction of c-Fos expression in APP^NL−G−F^ mice. On the other hand, a previous study showed elevated c-Fos levels in 3-month-old APP^NL−G−F^ mice [75]. This discrepancy may reflect change in temporal dynamics of affected neurons upon exposure to Aβ stress. Supporting this, c-Fos is rapidly induced in response to acute neuronal stimulation but suppressed under conditions of chronic activity [76]. Moreover, transcriptional repression of c-Fos is mediated by ΔFosB, a highly stable Fos-family protein that accumulates with chronic neuronal activation. Consistently, ΔFosB is upregulated in Aβ pathology models, and its inhibition restores c-Fos expression and ameliorates cognitive deficits [76]. Given that our analysis was performed in 5.5- to 6-month-old mice, an age at which Aβ pathology is more established [33], it is plausible that the observed c-Fos downregulation reflects chronic neuronal stress. Restoration of c-Fos expression by Kvβ2 expression suggests that Kvβ2 may counteract such chronic dysfunction, thereby preserving neuronal activity and cognitive function. Further works across different ages in APP^NL−G−F^ mice are necessary to understand the temporal regulation of c-Fos and ΔFosB during disease progression.

In APP^NL−G−F^ mice, We observed a reduction in the signal intensity of NeuN immunoreactivity rather than a decrease in the number of NeuN-positive neurons. In Aβ-accumulating Alzheimer’s disease models, reductions in NeuN levels are frequently observed. Several studies have reported that this decrease does not necessarily reflect a loss of NeuN-positive neurons or neuronal loss [77, 78]. NeuN signal reduction might occur under various neuropathological stress conditions, rather than exclusively reflecting neuronal loss [79]. One point we can speculate is that Aβ-binding to Kvβ2 induces Kvβ2 dysfunction, which might elicit neuropathological stress in neurons, leading to a reduction of NeuN signal. In APP^NL−G−F^ mice, the introduced Kvβ2 may bind to Aβ and thereby sequester Aβ away from endogenous Kvβ2, allowing the Kvβ2 protein to retain its physiological function. Therefore, Aβ-induced toxicity and neuropathological stress are likely alleviated through Kvβ2 expression in APP^NL−G−F^ mice, which may account for the rescue of NeuN signal intensity.

In summary, our findings reveal a previously unrecognized mechanism by which Aβ perturbs Kv1 channel function and highlight the crucial role of the Aβ-Kvβ2-Kv1 axis in cognitive deficits observed in Aβ pathology model mice. These results expand the current understanding of Aβ neurotoxicity and open new avenues for targeting ion channel dysregulation in Aβ pathology.

## Conclusion

This study identifies Kvβ2 as a novel intracellular binding partner of Aβ and elucidates its role in mediating Aβ-induced neurotoxicity. We demonstrate that Aβ directly binds to the N-terminus of Kvβ2, disrupting its interaction with EB1. This interference impairs the membrane trafficking of Kv1 channels, leading to reduced potassium channel activity, neuronal hyperexcitability, and cognitive deficits in APP^NL−G−F^ mice. Importantly, ectopic expression of Kvβ2 ameliorates Aβ-induced hyperexcitability and memory impairments, showing the functional relevance of the Aβ-Kvβ2 interaction. These findings reveal a previously unrecognized mechanism by which intracellular Aβ contributes to early-stage neuronal dysfunction by disrupting ion channel trafficking in Aβ pathology.

## Supplementary Information

Below is the link to the electronic supplementary material.

Supplementary Material 1

Supplementary Material 2

## Data Availability

All data generated or analyzed during this study are included in this published article and its supplementary information files.

## Abbreviations

Aβ: Amyloid-β
AD: Alzheimer’s disease
AIS: Axon initial segment
AnkG: Ankyrin G
Co-IP: Co-immunoprecipitation
EB1: End-binding protein 1
EPSC: Excitatory postsynaptic current
ICV: Intracerebroventricular
Kv: Voltage-gated potassium channel
Kvβ2: Voltage-gated potassium channel β2
MB: Methylene blue
NFA: Niflumic acid
PFA: Paraformaldehyde
PI: Propidium iodide
SPR: Surface plasmon resonance
WT: Wild type
