# Supplementary material for: Altered trafficking of Kv1-Kvβ2 leads to neuronal hyperexcitability and memory deficits in amyloid-β pathology
Source: Mol Neurodegener. 2026 Mar 10;21:22. doi: 10.1186/s13024-026-00936-2 (PMC13088556; doi:10.1186/s13024-026-00936-2)
Supplement: Supplementary file 1 — Supplementary Material 1 [file 13024_2026_936_MOESM1_ESM.docx]

**Altered Trafficking of Kv1-Kvβ2 Leads to Neuronal Hyperexcitability and Memory Deficits in Amyloid-β Pathology**

Revised version submitted to *Molecular Neurodegeneration*

Young-Sin Cho^1†^, Seo-Hyun Kim^1,2†^, Shin-Hyeon Ryu^1^, Chaelin Chung^3^, Nahyun Lee^1^, Namhoon Kim^4^, Muhah Jeong^1^, Youngwon Kim^1^, Jimin Gwak^1^, Se-Young Choi^3^, Takaomi Saido^5^, Yong-Keun Jung^1,4^*

^1^School of Biological Sciences, Seoul National University, Seoul 08826, Korea

^2^Department of Neurology and Institute for Cell Engineering, Johns Hopkins University School of Medicine, Baltimore, MD 21205, USA.

^3^Department of Physiology, Dental Research Institute, Seoul National University School of Dentistry, Seoul 03080, Korea

^4^Interdisciplinary Program in Neuroscience, Seoul National University, Seoul 08826, Korea

^5^Laboratory for Proteolytic Neuroscience, RIKEN Center for Brain Science, Saitama 3510198, Japan

^†^These authors equally contributed to this work.

*Corresponding author: (Y.-K.J.) School of Biological Sciences, Seoul National University, 1 Gwanak-ro, Gwanak-gu, Seoul 08826, Republic of Korea. Tel: +82-2-880-4401, E-mail: [ykjung@snu.ac.kr](mailto:ykjung@snu.ac.kr)

**Supplementary Figures**


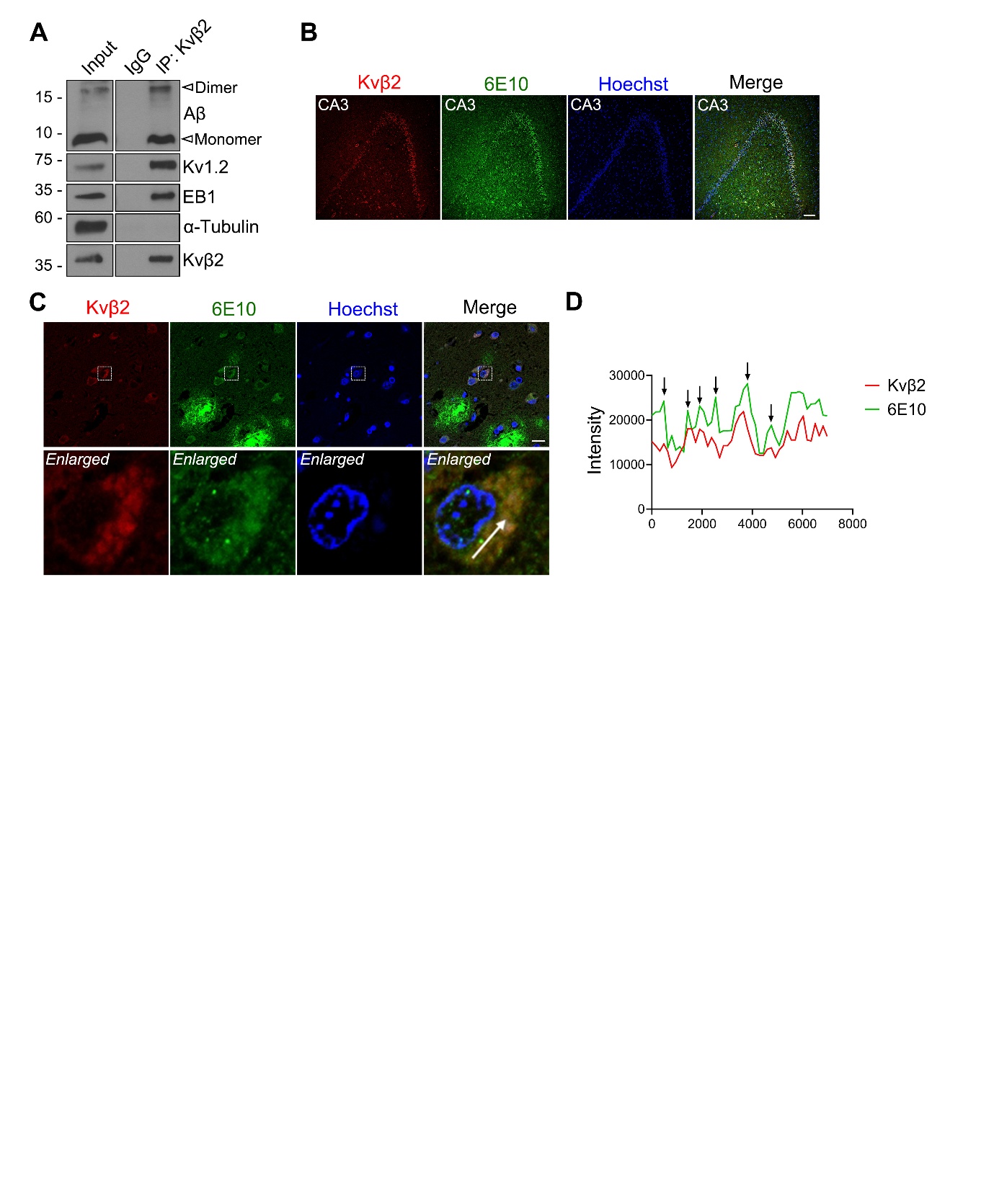
**Supplementary Fig. 1**

**A** The hippocampal lysates of 9-month-old APP^NL-G-F^ mice were subjected to immunoprecipitation (IP) assays using anti-Kvβ2 (Neuromab) antibody, followed by Western blot. The 10% of the total cell lysate was loaded as input. **B, C** Paraffin sections of the hippocampus from patients with AD were immunostained with anti-Kvβ2 (Origene) and 6E10 antibodies, and nuclei were stained using Hoechst33342 (Thermo Scientific, 62249). White line indicates the region of interest used for line-scan fluorescence intensity analysis. Scale bars, 100 μm **(B)** and 10 μm **(C)**. **(D)** The fluorescent intensities of Kvβ2 and 6E10 on the line in the enlarged images were measured using Zen software and positions showing coincident peaks in the intensity profiles were marked by arrows.


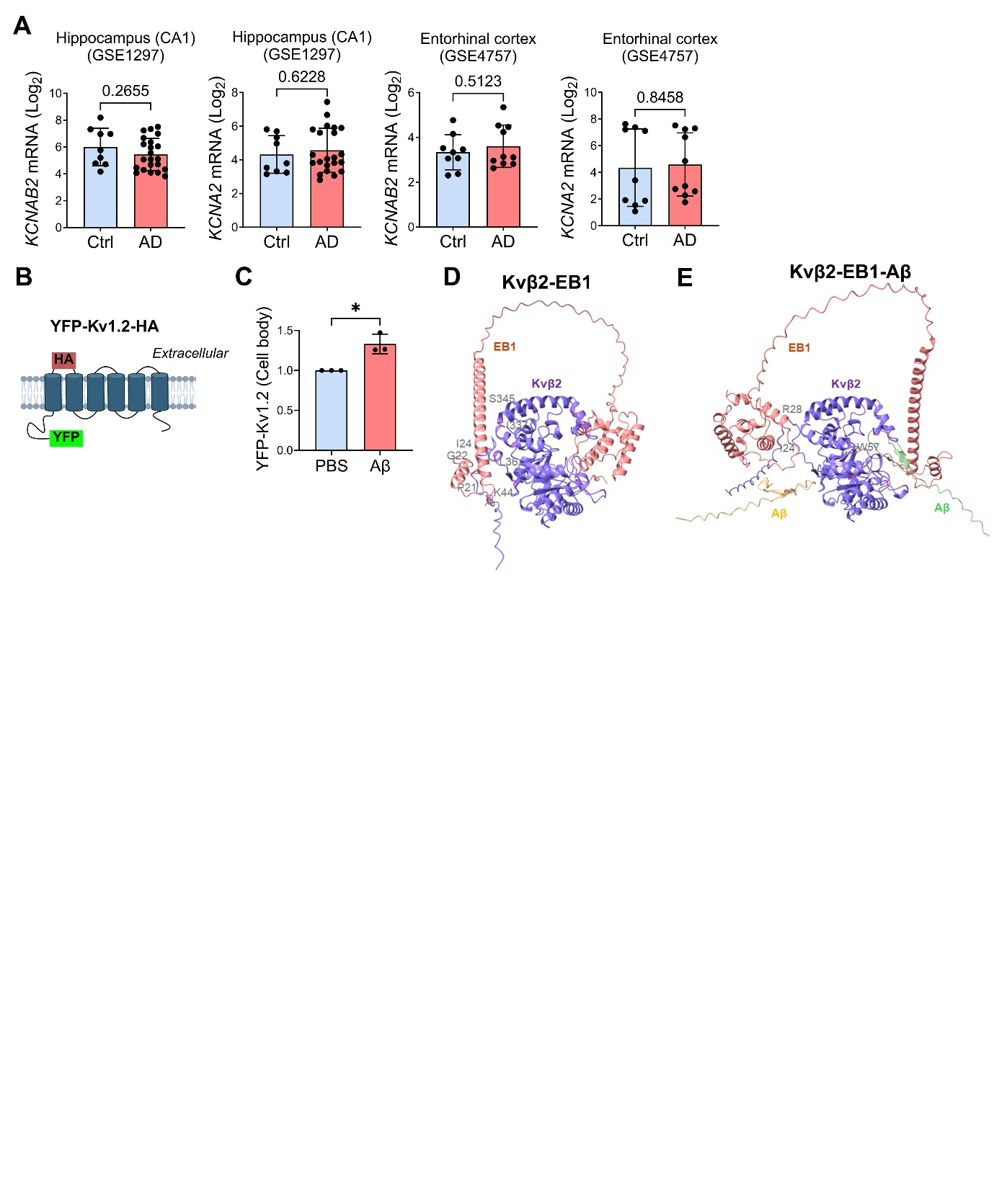
**Supplementary Fig. 2**

**A** *KCNAB2* and *KCNA2* mRNA levels were analyzed in microarray datasets of control and AD samples from the Gene Expression Omnibus, including GSE1297 (hippocampal CA1 region; control, *n* = 9; AD, *n* = 22) and GSE4757 (entorhinal cortex; control, *n* = 9; AD, *n* = 10). **B** Schematic representation of YFP-Kv1.2-HA fusion protein in which YFP is fused to the N-terminus and HA tag is inserted in the extracellular domain between transmembrane 1 and 2 of Kv1.2. **C** Primary cortical neurons (DIV 8-10) were transduced with lentiviruses carrying YFP-Kv1.2-HA for 72 h and treated with Aβ (5 μM) for additional 30 h. After immunostaining using anti-HA antibody without permeabilization, cells were observed under fluorescence microscope. YFP-Kv1.2 level in cell body was quantified using ImageJ (*n* = 3). **D, E** Predicted structures of the Kvβ2 (purple)-EB1 (red) complex in the absence **(D)** or presence **(E)** of monomeric Aβ (yellow and green), generated using AlphaFold. Amino acid residues within the N-terminal and C-terminal regions of Kvβ2 previously implicated in EB1-binding are highlighted (gray).

Bars represent mean ± S.D. Unpaired *t*-test, two-tailed **(A, C).** **P* < 0.05.


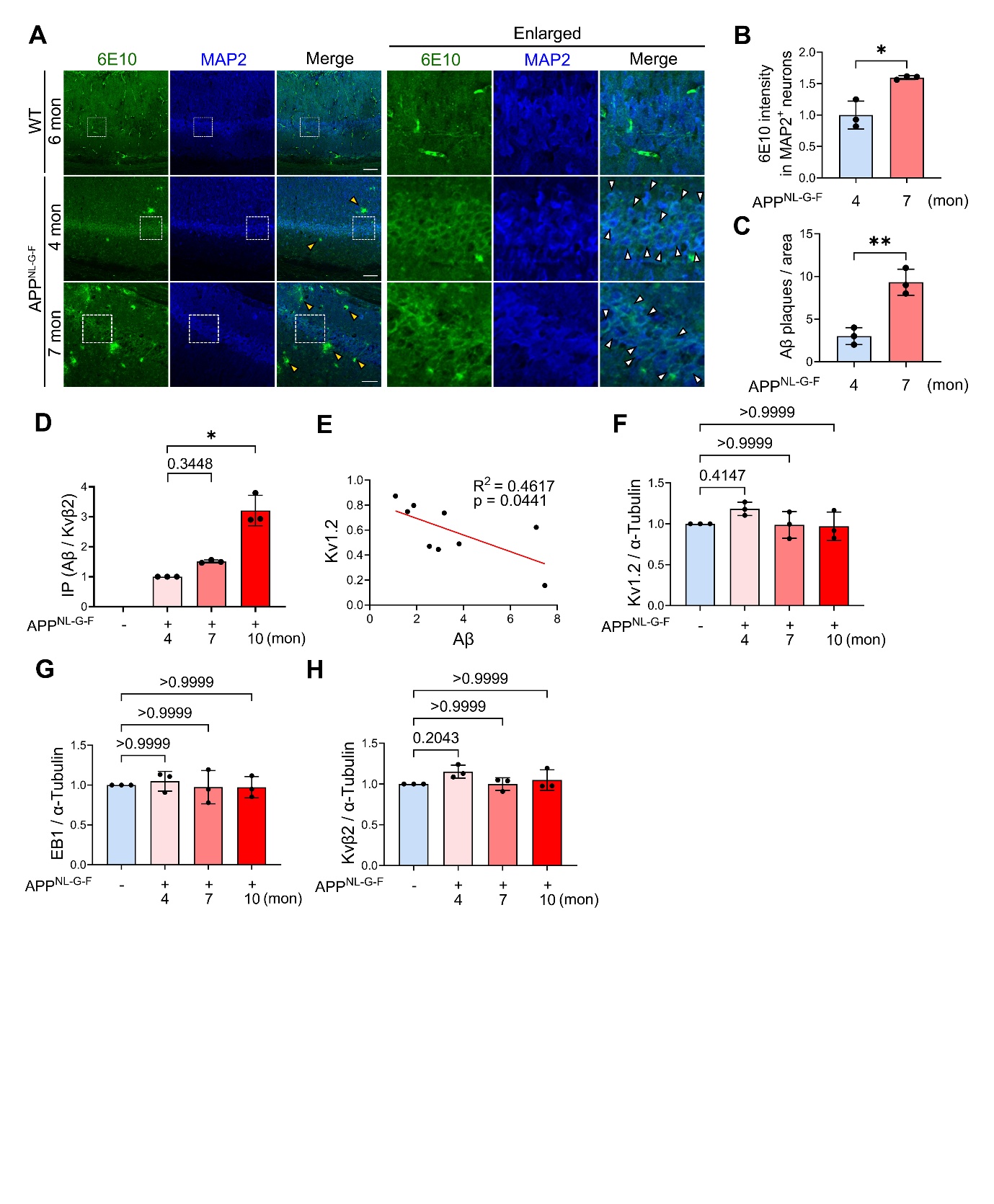
**Supplementary Fig. 3**

**A** Confocal images of the hippocampal CA1 region of 6-month-old wild-type (WT) and 4- and 7-month-old APP^NL-G-F^ mice immunostained with 6E10 and anti-MAP2 antibodies. Enlarged views correspond to the areas indicated by white boxes. Yellow arrowheads indicate Aβ plaques and white arrowheads indicate 6E10 signals within the MAP2-positive neurons. Scale bars, 100 μm. **B, C** Quantification of 6E10 signal intensity in the MAP2-positive neurons **(B)** and Aβ plaque density per unit area (*n* = 3) **(C)**. **D-H** Hippocampal lysates of 4-, 7-, and 10-month-old APP^NL-G-F^ mice and 6-month-old WT mice were subjected to IP assays using anti-Kvβ2 (Neuromab) antibody. Levels of the immunoprecipitated Aβ were normalized with the immunoprecipitated Kvβ2 **(D)**. The relationship between the immunoprecipitated Kv1.2 and immunoprecipitated Aβ levels was analyzed using linear regression, with an R square value and a *P*-value (*n* = 9). Statistical significance was determined using linear regression analysis **(E)**. Levels of Kv1.2 **(F)**, EB1 **(G)** and Kvβ2 **(H)** were normalized with α-tubulin (*n* = 3).

Bars represent mean ± S.D. Unpaired t-test, two-tailed **(B, C)**. One-Way ANOVA with Bonferroni post-hoc analysis **(D, F, G, H)**. **P* < 0.05, ***P* < 0.01.


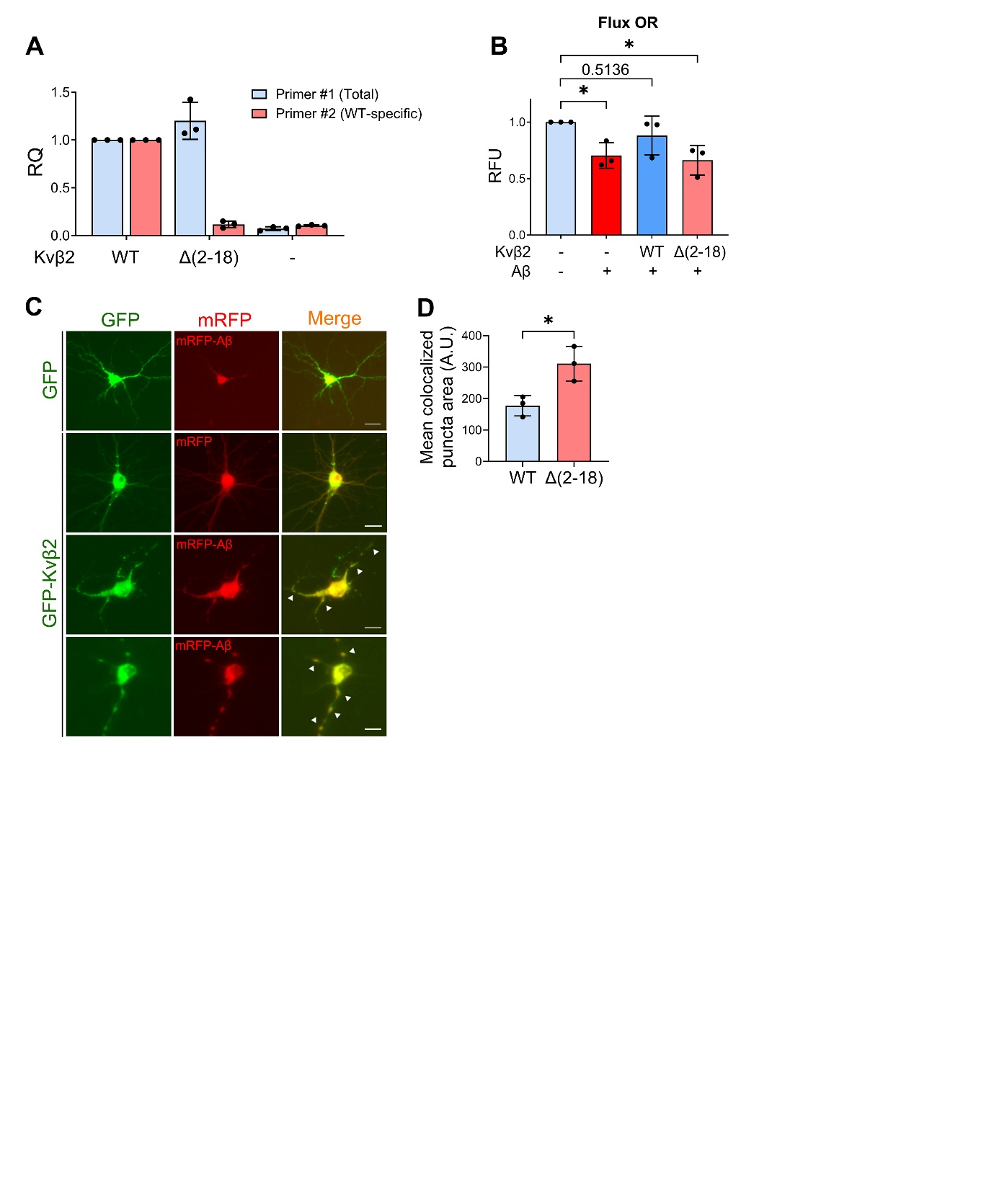
**Supplementary Fig. 4**

**A** Quantification of *KCNAB2* mRNA levels in HT22 cells expressing either Kvβ2 WT or Δ(2-18) mutant. Total RNAs were isolated from cells, followed by cDNA synthesis. SYBR Green-based quantitative PCR was performed using two independent primer pairs: One targeting a common region present in both Kvβ2 WT and Δ(2-18) mutant, and the other spanning the N-terminal region deleted in the Δ(2-18) mutant, thereby amplifying only WT transcript. Relative quantification (RQ) was conducted using the ΔΔCt method, with Gapdh as the reference gene (*n* = 3). Primer #1: 5′- AGC GTG AGA AAG TGG AGG TGC A -3′ and 5′- AAG GAG GCT CTT GAG TAG GGT G -3′, Primer #2: 5′- ACG ACG GGC TCC CCG GCT CGG C -3′ and 5′- GGC CAG ACT TGC CCA GGT TCC T -3′. **B** HT22 cells stably expressing Kvβ2 WT or Δ(2-18) mutant were treated with Aβ (5 μM) for 30 h, followed by an in vitro Flux OR assay to measure potassium channel activity (*n* = 3). **C** Primary hippocampal neurons (DIV 12) were transfected with GFP-C1 or GFP-Kvβ2 together and mRFP-C1 or mRFP-Aβ using Lipofectamine reagent (Invitrogen, 52887) for 36 h and observed under a fluorescence microscope. Arrowheads indicate the colocalization between GFP-Kvβ2 (green) and mRFP-Aβ (red) signals. Scale bars, 10 μm. **D** HT22/mRFP-Aβ cells were transfected with GFP-Kvβ2 WT or Δ(2-18) mutant for 24 h and observed under fluorescence microscope. The size distribution of mRFP-Aβ puncta colocalizing with GFP-Kvβ2 WT (*n* = 51 puncta) or Δ(2-18) mutant (*n* = 44 puncta) was quantified by image J.

Bars represent mean ± S.D. Unpaired t-test **(D)**. One-Way ANOVA with Bonferroni post-hoc analysis **(B).** **P* < 0.05.


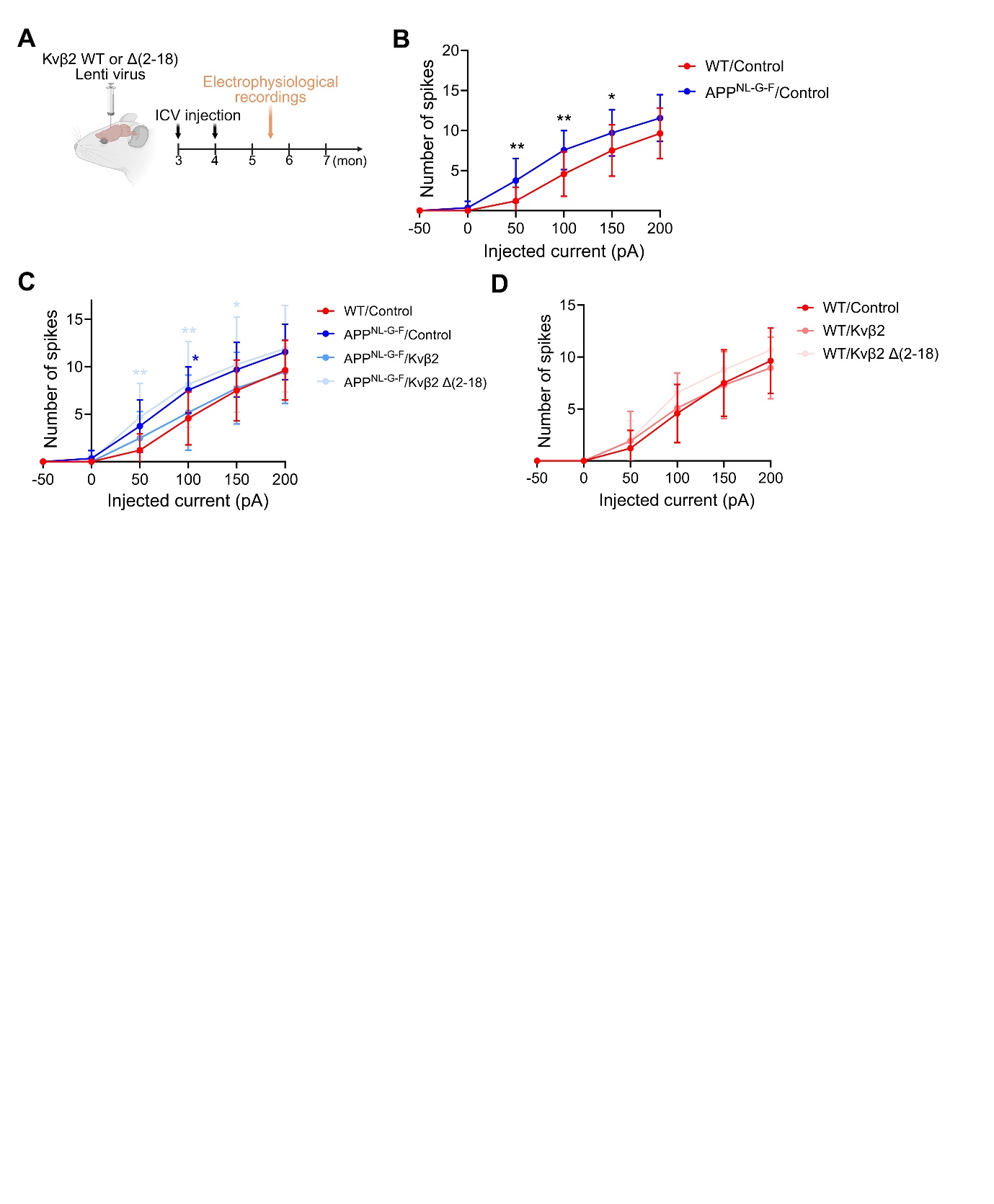
**Supplementary Fig. 5**

**A** Experimental strategy showing ICV injection of lentivirus expressing Kvβ2 WT or Δ(2-18) mutant and electrophysiological recordings in APP^NL-G-F^ mice. **B-D** Input-output curves were compared among the following groups: WT/Control, APP^NL-G-F^/Control **(B)**; WT/Control, APP^NL-G-F^/Control, APP^NL-G-F^/Kvβ2, and APP^NL-G-F^/Kvβ2 Δ(2-18) **(C)**; WT/Control, WT/Kvβ2, and WT/Kvβ2 Δ(2-18) **(D)**. Action potential frequency was measured in response to injected currents of -50, 0, 50, 100, 150, and 200 pA.

Data points represent mean ± S.D. Two-Way ANOVA with Bonferroni post-hoc analysis **(B, C, D)**. **P* < 0.05, ***P* < 0.01.


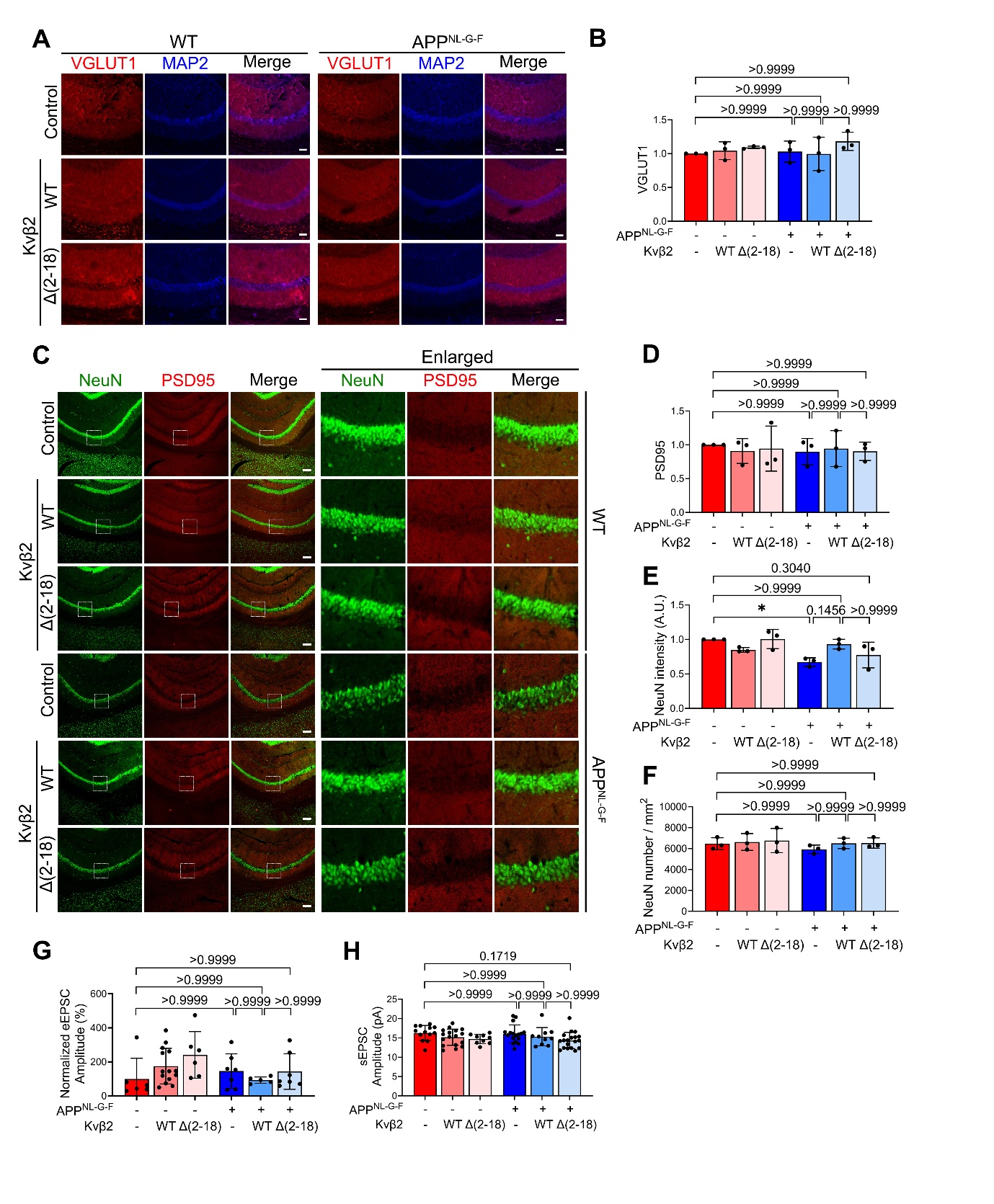
**Supplementary Fig. 6**

**A-F** Hippocampal CA1 sections obtained from WT and APP^NL-G-F^ mice expressing Kvβ2 WT or Kvβ2 Δ(2-18) mutant were subjected to immunostaining assay to detect VGLUT1 and MAP2 **(A),** and NeuN and PSD95 **(C).** Scale bars, 100 μm. The immunofluorescence signals of VGLUT1 **(B)**, NeuN **(E, F)**, and PSD95 **(D)** were quantified using ImageJ. **G, H** The hippocampal tissues slices were assessed for electrophysiological recordings to measure eEPSC **(G)** and sEPSC **(H)** amplitude. WT/Control: *n* = 6 cells, WT/Kvβ2: *n* = 14 cells, WT/Kvβ2 Δ(2-18): *n* = 6 cells, APP^NL-G-F^/Control: *n* = 7 cells, APP^NL-G-F^/Kvβ2: *n* = 5 cells, APP^NL-G-F^/Kvβ2 Δ(2-18): *n* = 7 cells.

Bars represent mean ± S.D. Two-Way ANOVA with Bonferroni post-hoc analysis **(B, D, E, F, G, H)**. **P* < 0.05.


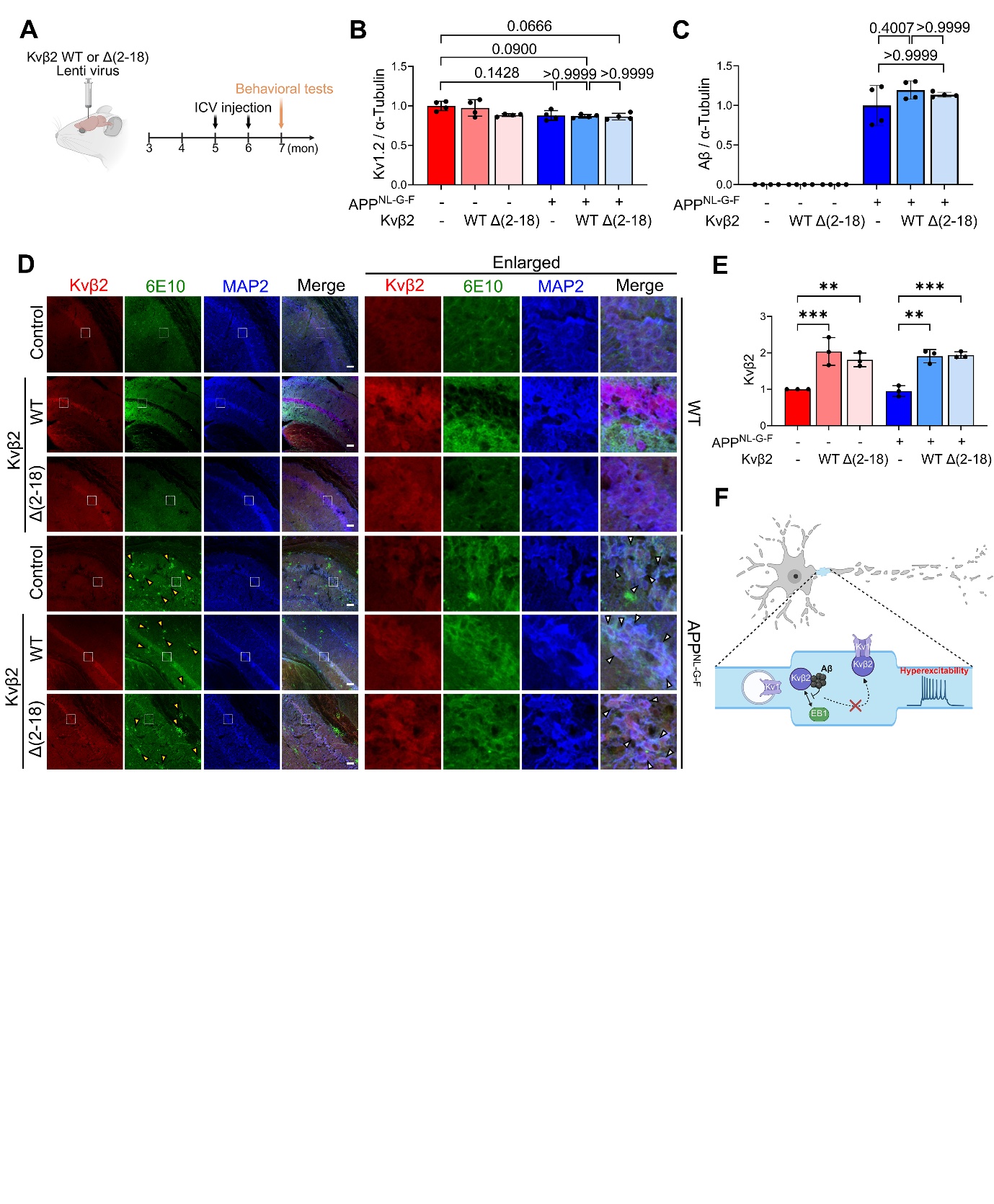
**Supplementary Fig. 7**

**A** Experimental strategy showing ICV injection of lentivirus expressing Kvβ2 WT or Δ(2-18) mutant and behavioral tests in APP^NL-G-F^ mice. **B, C** Hippocampal tissue lysates of 5.5- to 6-month-old mice were subjected to Western blot analysis. The levels of Kv1.2 **(B)** and Aβ **(C)** on the blots were quantified using ImageJ and normalized by α-Tubulin (*n* = 4). **D** Representative confocal images of the hippocampal CA1 region from cryosections of the mouse groups used in in vivo experiments, immunostained with anti-Kvβ2 (Origene), 6E10, and anti-MAP2 antibodies. Enlarged views correspond to the areas indicated by white boxes. Yellow arrowheads indicate Aβ plaques and white arrowheads indicate Kvβ2-expressing neurons that are co-distributed with intraneuronal Aβ signals inside MAP2-positive neurons. Scale bars, 20 μm. **E** The immunofluorescence signals of Kvβ2 were quantified using ImageJ (*n* = 3). **F** The proposed model shows how Aβ disrupts the Kv1-Kvβ2-EB1 complex via binding to Kvβ2, leading to mis-localization of Kv1 and subsequent neuronal dysfunction in Aβ pathology.

Bars represent mean ± S.D. Two-Way ANOVA with Bonferroni post-hoc analysis **(B, C, E)**. ***P* < 0.01, ****P* < 0.001.

**Supplementary file: Uncropped Western blot images**

Due to the use of multiple antibodies on the same blot, specific lanes were cropped for clarity and presentation in the main figures and supplementary figures. No image enhancement or data manipulation was performed, and all presented data accurately reflect the original blot results. Each uncropped Western blot image is labeled with the corresponding figure and panel number.


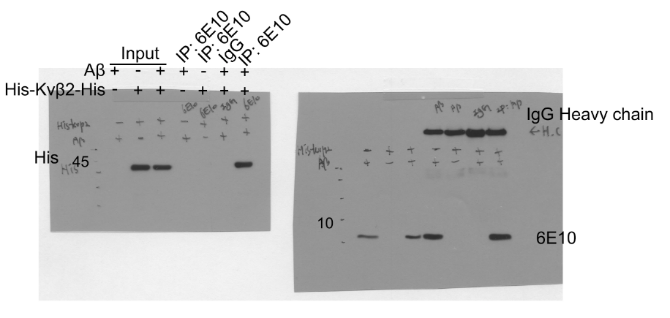


**Fig. 1E**


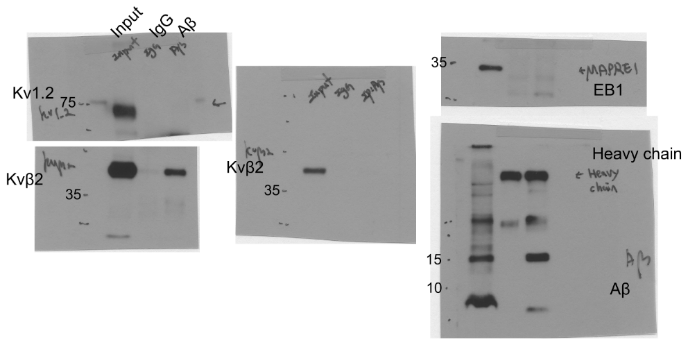


**Fig. 1C**


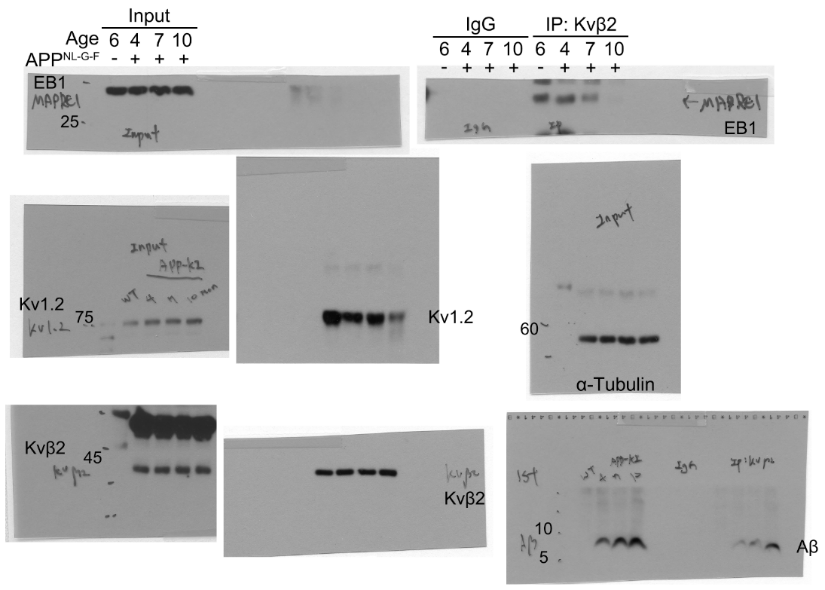


**Fig. 2E**

**Fig. 2E**

**Fig. 2H**


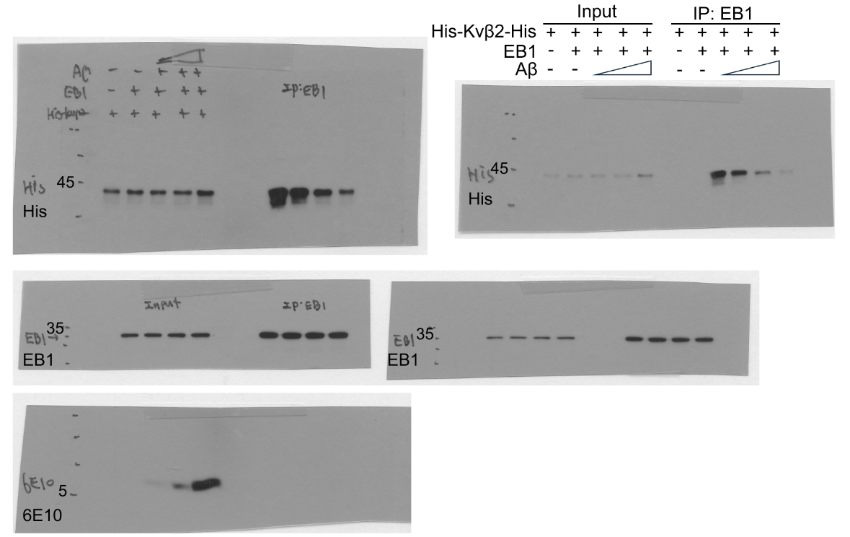


**Fig. 2H**


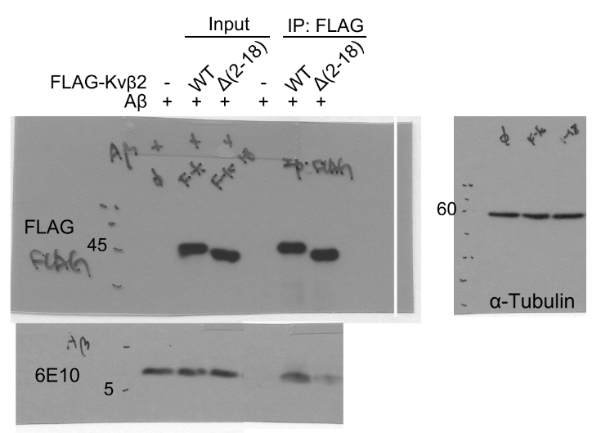


**Fig. 3C**


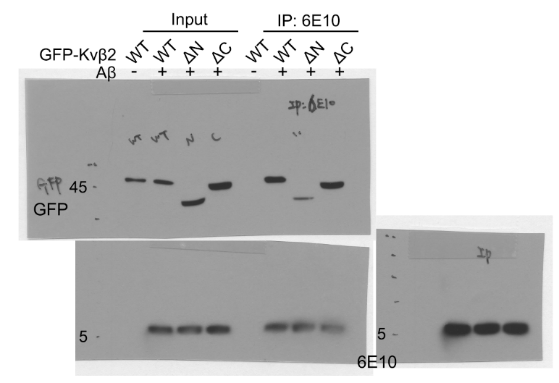


**Fig. 3B**

**Fig. 3E**


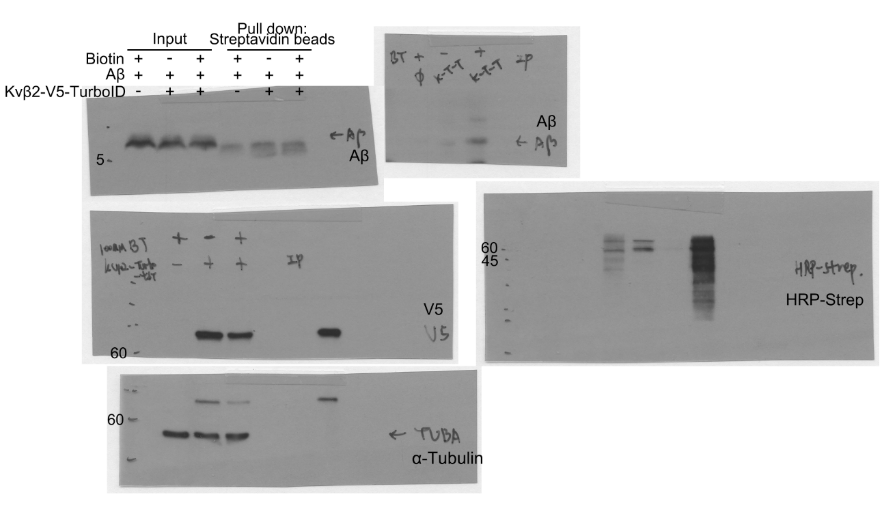

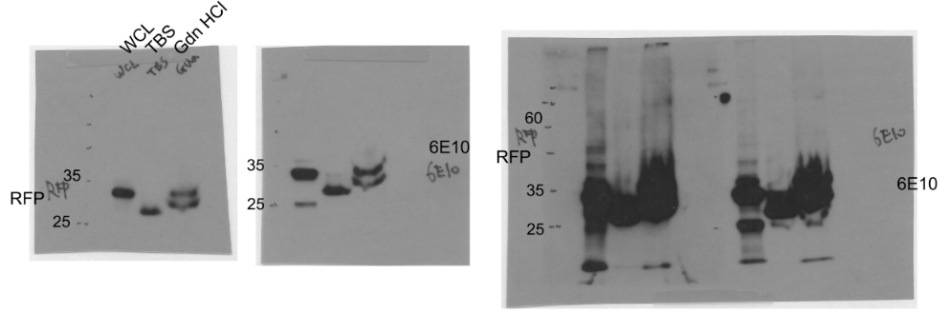


**Fig. 4A**


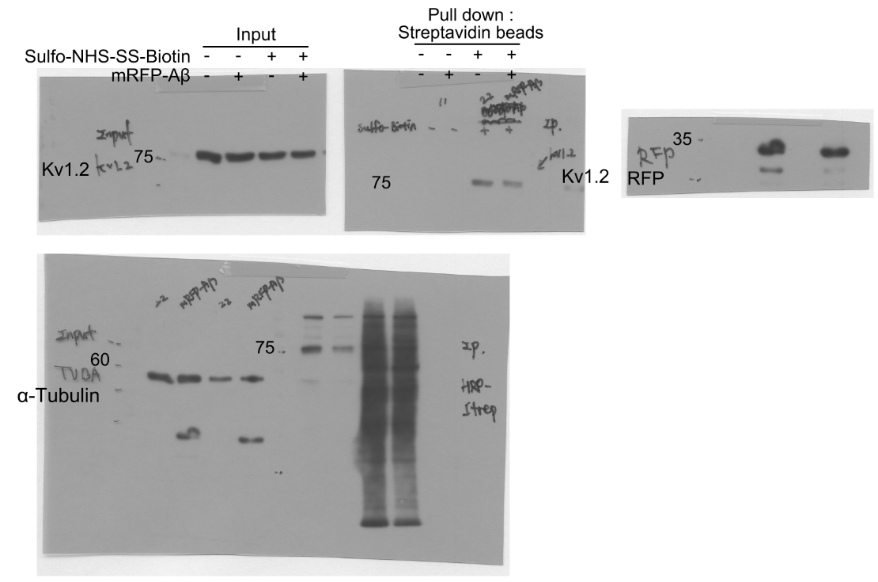


**Fig. 4K**


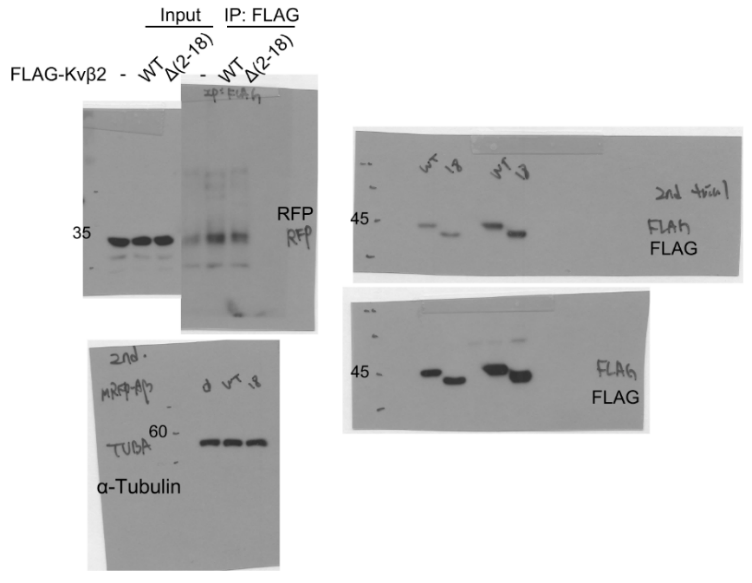


**Fig. 4I**


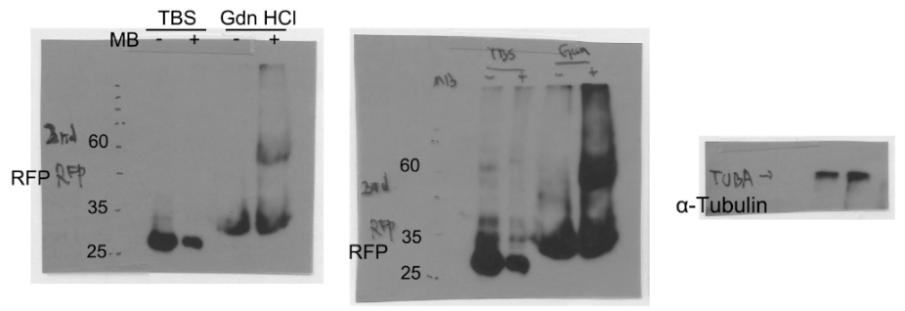


**Fig. 4E**


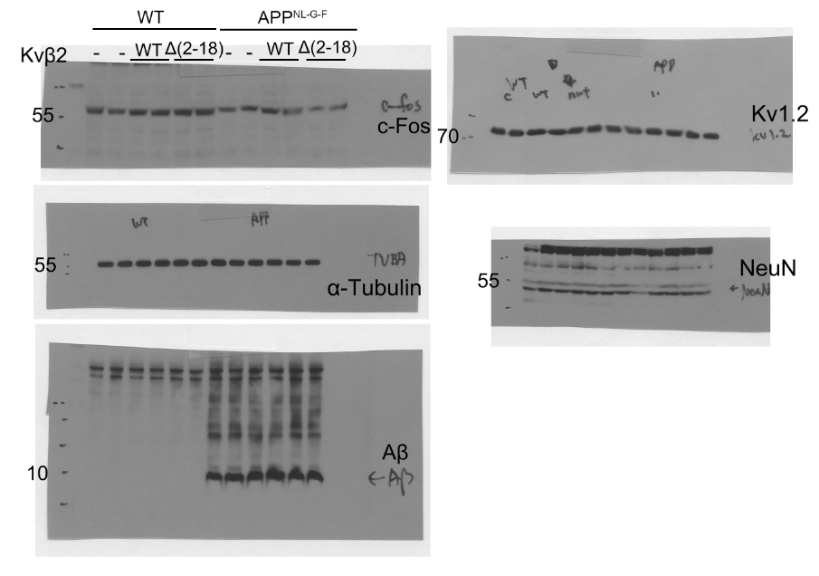


**Fig. 5H**

**
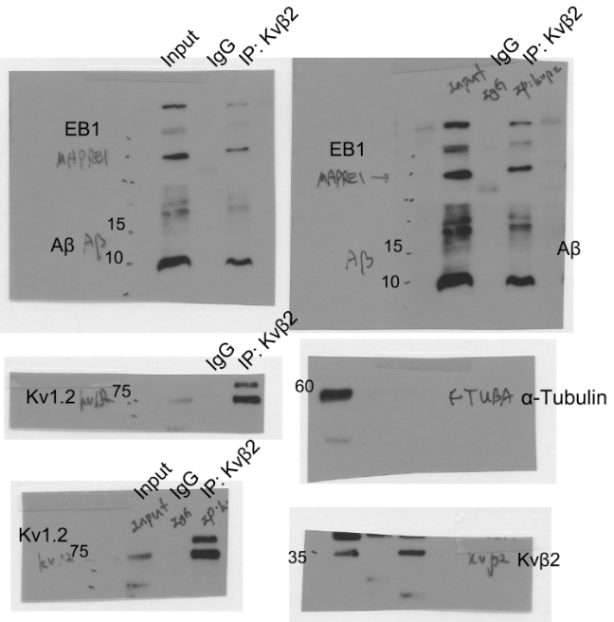
**

**Supplementary Fig. 1A**
